# Bambusurils as a mechanistic tool for probing anion effects†

**DOI:** 10.1039/c9fd00038k

**Published:** 2019-06-04

**Authors:** Lucie Jašíková, Maitê Rodrigues, Jana Lapešová, Tomáš Lízal, Vladimír Šindelář, Jana Roithová

**Affiliations:** Faculty of Science, Charles University in Prague Hlavova 2030 12843 Prague 6 Czech Republic; T. G. Masaryk Water Research Institute p. r. i., Podbabská 30 Prague 6 Czech Republic; Department of Chemistry, RECETOX, Faculty of Science, Masaryk University Kamenice 5 625 00 Brno Czech Republic; Institute for Molecules and Materials, Radboud University Heyendaalseweg 135 6525 AJ Nijmegen Netherlands jana.roithova@ru.nl

## Abstract

Bambusuril macrocycles have high affinity towards anions (X^−^) such as PF_6_^−^ and SbF_6_^−^ or BF_4_^−^ and ClO_4_^−^. Therefore, addition of bambusurils to reaction mixtures containing these anions effectively removes the free anions from the reaction process. Hence, comparing reactions with and without addition of bambusurils can demonstrate whether the anions actively participate in the reaction mechanism or not. We show this approach for gold(i) mediated addition of methanol to an alkyne. The reaction mechanism can proceed *via* monoaurated intermediates (*e.g.*, in catalysis with [(IPr)AuX]) or *via* diaurated intermediates (*e.g.*, in catalysis with [(PPh_3_)AuX]). We show that anions X^−^ slightly affect the reaction rates, however the effect stays almost the same even after their encapsulation in the cavity of bambusurils. We also demonstrate that X^−^ affects the overall reaction rate in the very same way as the reaction rate of the protodeauration step. All results are consistent with the indirect effect of X^−^ by the acidity of the conjugated acid HX on the rate-determining step. There is no evidence that a direct involvement of X^−^ would affect the reaction rate.

## Abstract 2

We present bambusurils, macrocycles which strongly bind anions of different sizes, as a mechanistic tool to probe counter ion effects in metal catalysis.

## Graphical abstract

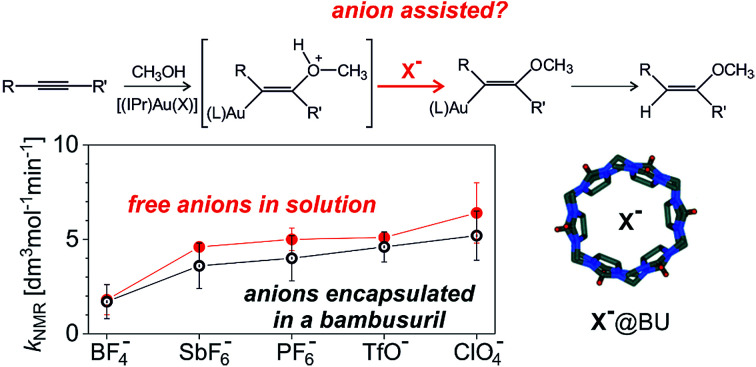

## Introduction

Many organometallic reactions are catalysed by a cationic complex. Examples include reactions catalysed by ruthenium-,^1^ rhodium-,^2^ iridium-,^3^ gold-^4^ and other complexes.^5^ In considering and suggesting reaction mechanisms, we pay most attention to cationic complexes involving the ionized catalysts and tend to neglect the role of anions. The anions are often designed so they do not directly bind to the metal centre and so they play an observant role in the reaction.^1–5^ Typical anions of this kind are PF_6_^−^, SbF_6_^−^ or BF_4_^−^. However, some cationic catalysts are used as salts with anions such as triflate (TfO^−^), trifluoroacetate, acetate or tosylate that can actively participate in the reaction. Mostly, they are involved in proton shuttling steps^6–8^ or in deprotonation of the substrate.^9,10^

The anion effect on the catalysed reaction is usually evaluated from a comparison of the rates of reactions catalysed with the same cationic catalyst, but with altered counter ions.^11,12^ The effect of the anion can however stem not only from its active participation in reaction mechanisms, but also from forming the conjugated acid and thus affecting the pH of the reaction mixture. One of the effects can be dominating and the other negligible. However, there are probably reactions in which the effects are in synergy or antagonism. Hence, it is important to have tools to evaluate these effects and to understand them.

**Scheme 1:**
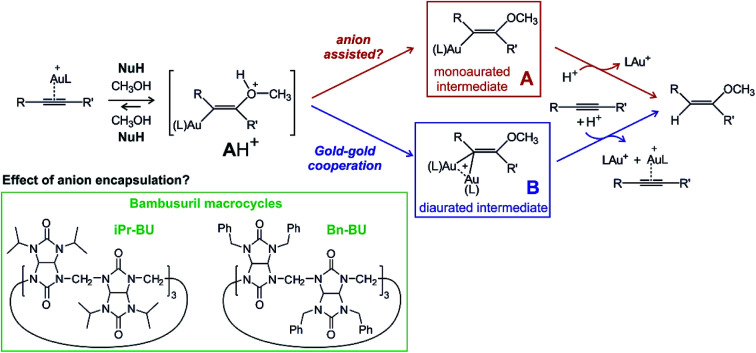
Proposed mechanism for nucleophilic addition to alkyne catalysed by gold(i) and the structures of the studied bambusurils.

An example of a reaction where the anion might or might not play an active role is gold(i) mediated nucleophilic addition to alkynes.^13,14^ Numerous mechanistic studies of different variants of this reaction identified two reaction mechanisms proceeding *via* two different key intermediates (Scheme 1). The first path leads through the addition of nucleophile NuH to the gold-activated triple bond forming a transient cationic intermediate [**A**H]^+^. [**A**H]^+^ gets deprotonated and forms the key monoaurated intermediate **A**.^15,16^ The second, dual activation pathway, involves the gold-activated nucleophile [(Nu)Au(L)] (L is a ligand of the gold(i) catalyst) which attacks the gold-activated triple bond. The key intermediate is thus diaurated complex **B**.^17–21^ The rate-determining step for both types of mechanisms is protodeauration of the key intermediates and therefore, the reaction is accelerated in the presence of acids.^22^

The need for an acid for protodeauration and the precondition of the deprotonation step on the single-activation pathway ([**A**H]^+^ → **A**) creates a playground for possible anion effects in the reaction mechanism.^23^ In fact, numerous studies suggested that the deprotonation step is mediated by the counter ions present in solution.^11,24^ The experimental rates of these reactions slightly change on changing the non-coordinating counter ions.^12^ Indeed, addition of coordinating counter-ions that do not detach from the gold complex can hamper or completely hinder these reactions, because the cationic gold is not available any more for the catalysis.^12^

A possible way to evaluate the role of anions is to compare reactions with free anions and with anions deactivated by selective encapsulation. Bambusurils are macrocyclic ligands that strongly bind anions in a 1 : 1 molar ratio.^25,26^ Anions are stabilized inside the bambusuril cavity by twelve C–H⋯anion hydrogen bonding interactions. The bambusuril framework is flexible and thus accepts anions of various sizes ranging from small F^−^ up to spacious SbF_6_^−^.^27^ Encapsulation of different anions into bambusuril macrocycles induces unique chemical shifts in ^1^H NMR spectra, and therefore can be qualitatively as well as quantitatively monitored.^27^ Herein, we propose and evaluate the use of bambusurils to test the anion effects in reaction mechanisms.

## Experimental

The active catalyst [Au(PPh_3_)X] was prepared by mixing 1 equiv. [Au(PPh_3_)Cl] and 1.2 equiv. AgX (X = BF_4_, SbF_6_, OTf, PF_6_, ClO_4_) in methanol (either CH_3_OH or CD_3_OH(D)). The AgCl precipitate was filtered off (PTFE filter, pore size 0.2 μm). The kinetics measurements were performed with 0.6 M 1-phenylpropyne and 1.25 mol% [Au(PPh_3_)X] in methanol with addition of different amounts of bambusurils. The exact compositions of all investigated reaction mixtures are in the ESI.† The MS experiments were performed with the same reaction mixtures, but 10 times diluted. Note that we have previously evaluated the effect of an added silver salt to these reaction mixtures and no effect on kinetics was detected whatsoever.^20^

**Scheme 2:**
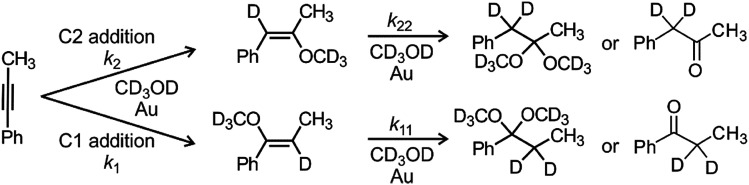
Addition of methanol to 1-phenylpropyne. Modelling of the time evolution of the product signals in NMR spectra provides rate constants for all individual steps (*k*_1_, *k*_2_, *k*_11_, *k*_22_, see Table 1).

The NMR experiments were recorded using a Varian NMR System (300 MHz) and the *δ* scale was referenced to the solvent residual peak at *δ* = 3.31 ppm. We used toluene as an internal standard. We mixed the solutions of a catalyst and reactants and immediately probed using the NMR instrument. The representative ^1^H NMR spectra can be found in the ESI (Fig. S3–S13†). 1-Phenylpropyne is converted initially to 1- or 2-methoxy-1-phenylpropene and finally to 1,1- or 2,2-dimethoxy-1-phenylpropane which hydrolyse to ethylphenylketone or benzylmethylketone, respectively (Scheme 2). We have integrated the corresponding peaks as a function of the reaction time and fitted the results with kinetic equations describing the reaction in Scheme 2. All equations and details of kinetic modelling can be found in the ESI.†

The mass spectrometry experiments were performed with a LTQ XL, three-segment 2D linear ion trap mass spectrometer. The ions were generated by electrospray ionization (ESI) at soft ionization conditions (low potentials on the entrance ion optics). The temperature of the capillary was 275 °C. The kinetics of diaurated intermediates in solution were determined by the delayed reactant labelling method. This method is based on monitoring of a reaction mixture containing isotopically unlabelled and labelled molecules, one of them being added with a time delay. The mutual evolution of peak intensities of the complexes containing unlabelled and labelled reactants, respectively, relates to the reaction kinetics in solution. The details are in the ESI† and in ref. 20.

## Results and discussion

The prototypical gold-mediated nucleophilic addition is addition of alcohol to alkyne. We have shown that with trimethylphosphino gold or triphenylphosphino gold catalyst, the addition proceeds *via* the diaurated reaction pathway (blue in Scheme 1). Gold with *N*-heterocyclic carbene ligand IPr (IPr = 1,3-bis(2,6-diisopropylphenyl)imidazol-2-ylidene)^28^ catalyses the reaction through the monoaurated path (red in Scheme 1). It has been suggested that the anions present in solution participate in deprotonation of the initial **A**H^+^ adduct.^11,12^ In order to investigate this possibility, we have monitored kinetics of methanol addition to 1-phenylpropyne catalysed by [(PPh_3_)AuX] or [(IPr)AuX] (X = BF_4_, SbF_6_, PF_6_, TfO, ClO_4_) with NMR and tested the effect of encapsulation of the anions by bambusuril macrocycles. We have also tested the effect of increasing the acidity of the solution in combination with encapsulation of the anions. The gold salts were prepared by anion exchange from the gold chlorides and the corresponding silver salt (see the Experimental section).

We have chosen two types of bambusuril macrocycles, dodecabenzylbambusuril (Bn-BU) and dodecaisopropylbambusuril (iPr-BU). The binding constants *K*_a_ in CHCl_3_ of X^−^@Bn-BU are 2.1 × 10^5^ for TfO^−^, 2.6 × 10^5^ for SbF_6_^−^, 8.7 × 10^8^ for PF_6_^−^, 1 × 10^10^ for BF_4_^−^ and 2.1 × 10^10^ for ClO_4_^−^.^27^ Hence, the relative concentration of free anions drops by 97% up to by 99.99% after addition of 1 equivalent of the bambusuril to the reaction mixture. Changes in reaction kinetics after the addition of bambusuril could be thus directly correlated to the role of anion in the reaction mechanism.

The iPr-BU bambusuril was prepared as a protonated complex with encapsulated HSO_4_^−^ (iPr-BU*H_2_SO_4_) for competition experiments. The HSO_4_^−^ anion has a medium binding affinity to bambusurils (for comparison, *K*_a_ in CHCl_3_ of HSO_4_^−^@Bn-BU is 3.5 × 10^8^).^27^ We have investigated all anions addressed here in competition experiments with HSO_4_^−^@iPr-BU in methanol (Fig. S20 and S21†) by NMR spectroscopy. Under the conditions of our experiments, addition of iPr-BU*H_2_SO_4_ to a solution of [(L)AuX] leads to quantitative encapsulation of BF_4_^−^, ClO_4_^−^, and PF_6_^−^ and to concomitant release of one equivalent of HSO_4_^−^. For TfO^−^ and SbF_6_^−^ we observed a rapid exchange of anions in the cavity of iPr-BU.

The experiments with iPr-BU and Bn-BU had to be performed in different solvent mixtures because of solubility problems. The experiments with Bn-BU were performed in a 1 : 2.4 mixture of methanol and dichloromethane, whereas the experiments with iPr-BU*H_2_SO_4_ were done in a 1 : 0.08 ratio of methanol and dichloromethane. Accordingly, the reference reactions without addition of bambusurils are slightly different for both sets of reactions.

### Reaction pathway proceeding through monoaurated intermediate A (catalysis by (IPr)Au^+^)

**Fig. 1:**
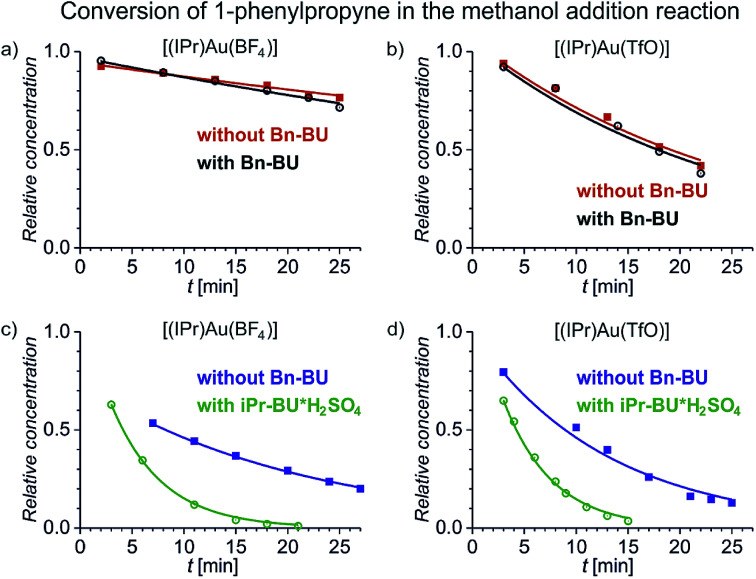
Conversion of 1-phenylpropyne to products in the methanol addition reaction catalysed by [(IPr)Au(BF_4_)] (a and c) and [(IPr)Au(TfO)] (b and d) (the increase in the concentration of the addition products and the results for the remaining investigated catalysts are in Fig. S1†). The experiments were performed in a mixture of methanol and dichloromethane: 1 : 2.4 (red, black) and 1 : 0.08 (blue, green), respectively. The hollow points refer to the experiment with addition of 1 equivalent of Bn-BU (black) or iPr-BU@H_2_SO_4_ (green) with respect to the concentration of the gold catalyst. The solid lines correspond to the fits using the kinetic scheme shown in Scheme 2 and described in the ESI.†

We have first investigated the effect of counter ion X^−^ on the overall kinetics of methanol addition to 1-phenylpropyne catalysed by [(IPr)AuX]. We have monitored the reaction by NMR spectroscopy (Fig. 1 and S1–S12†). As detailed in the previous studies, the reaction starts with addition of the first molecule of methanol to either the C1 or C2 carbon atom with rate constants *k*_1_ and *k*_2_ (see Scheme 2). Then it continues by the second addition of methanol with rates *k*_11_ and *k*_22_; the second addition of methanol to the C2 carbon atom is very fast, therefore the rate constants cannot be determined with sufficient precision (Table 1). We will further discuss only the overall rate of the reaction *k*_NMR_ which equals *k*_1_ + *k*_2_, but we provide all rate constants for possible future scrutiny in Table 1. The branching ratio for the C1 and C2 additions stays the same within the experimental error. The evolution of the concentration of the C1 and C2 addition products is in Fig. S1.†Fig. 1 shows only the decrease in concentration of 1-phenylpropyne resulting from its conversion to the products. The fits of the data in Fig. 1 give directly the *k*_NMR_ = (*k*_1_ + *k*_2_) rate constant.

**Table 1:** Rate constants obtained by NMR spectroscopy

Catalyst: [Au(IPr)X]^a^		*k*_NMR_ = *k*_1_ + *k*_2_	*k*_1_ (dm^3^ mol^−1^ min^−1^)	*k*_2_ (dm^3^ mol^−1^ min^−1^)	*k*_11_ (dm^3^ mol^−1^ min^−1^)	*k*_22_ (dm^3^ mol^−1^ min^−1^)
X	BU					
OTf^a^	—	11.8 ± 1.5	3.2 ± 1.0	8.7 ± 0.6	1.6 ± 0.8	65 ± 49
OTf^a^	iPr-BU*H_2_SO_4_	26.5 ± 1.6	7.5 ± 1.7	18.9 ± 3.5	4.7 ± 3.1	846 ± 759
ClO_4_^a^	—	11.2 ± 2.2	3.1 ± 1.0	8.0 ± 1.1	1.5 ± 0.6	92 ± 77
ClO_4_^a^	iPr-BU*H_2_SO_4_^a^	24.5 ± 3.4	6.2 ± 1.9	18.2 ± 1.4	3.1 ± 0.8	189 ± 99
PF_6_^a^	—	10.9 ± 1.5	2.8 ± 0.7	8.0 ± 0.7	1.2 ± 0.4	100 ± 85
PF_6_^a^	iPr-BU*H_2_SO_4_^a^	22.1 ± 0.6	5.7 ± 1.0	16.5 ± 1.5	3.6 ± 1.0	571 ± 515
BF_4_^a^	—	7.6 ± 1.4	2.2 ± 0.6	4.1 ± 0.6	0.1 ± 0.1	1.8 ± 0.1
BF_4_^a^	iPr-BU*H_2_SO_4_^a^	24.8 ± 2.9	7.3 ± 1.4	17.2 ± 1.2	3.0 ± 0.9	59 ± 2
SbF_6_^a^	—	12.0 ± 2.8	3.1 ± 1.0	8.8 ± 1.8	1.1 ± 0.1	100 ± 81
SbF_6_^a^	iPr-BU*H_2_SO_4_^a^	28.8 ± 0.7	7.2 ± 0.6	21.6 ± 0.9	2.7 ± 0.2	105 ± 38
OTf^b^	—	5.1 ± 0.1	1.5 ± 0.2	3.6 ± 0.3	0.6 ± 0.6	485 ± 240
OTf^b^	Bn-BU^b^	4.6 ± 0.8	1.3 ± 0.1	3.3 ± 0.8	0.6 ± 0.6	405 ± 231
ClO_4_^b^	—	6.4 ± 1.6	1.6 ± 0.1	4.7 ± 1.4	0.9 ± 0.9	578 ± 531
ClO_4_	Bn-BU^b^	5.2 ± 1.3	1.3 ± 0.1	3.9 ± 1.1	0.7 ± 0.7	403 ± 307
PF_6_^b^	—	5.0 ± 0.6	1.6 ± 0.4	3.5 ± 0.2	1.9 ± 0.1	1654 ± 1335
PF_6_	Bn-BU^b^	4.0 ± 0.5	1.2 ± 0.1	2.8 ± 0.5	0.6 ± 0.6	796 ± 630
BF_4_^b^	—	1.8 ± 0.8	1.0 ± 0.3	0.8 ± 0.5	0.1 ± 0.1	791 ± 761
BF_4_	Bn-BU^b^	1.7 ± 0.2	0.9 ± 0.2	0.9 ± 0.1	0.1 ± 0.1	197 ± 132
SbF_6_^b^	—	4.6 ± 0.1	1.4 ± 0.2	3.2 ± 0.1	1.5 ± 0.1	779 ± 452
SbF_6_	Bn-BU^b^	3.6 ± 0.2	1.2 ± 0.1	2.5 ± 0.2	0.6 ± 0.6	759 ± 567
Cl	Bn-BU^b^^,^^c^		No reaction			

a CD_3_OD : CD_2_Cl_2_ (1 : 0.08): 1.25 mol% [Au(IPr)X] with or without 1.25 mol% iPr-BU*H_2_SO_4_.

b In CD_3_OD : CD_2_Cl_2_ (1 : 2.4): 1.25 mol% [Au(IPr)X] with or without 1.25 mol% Bn-BU.

c The solution of Au(IPr)Cl and Bn-BU was mixed for 24 hours.

Fig. 1 demonstrates several key findings that we first shortly describe and then analyse in more detail below. Firstly, encapsulation of counter ions does not significantly affect the reaction rate of the addition reaction (compare the black and the red data). Secondly, the reaction rate slightly depends on the nature of the counter ion in [(IPr)AuX] regardless of whether the counter ion is encapsulated or not (compare panels *a* and *b* in Fig. 1). Thirdly, polarity/proticity of the solvent affects the rate of the addition reaction more than the counter ion in the catalysts [(IPr)AuX] (compare the red and the blue data in Fig. 1). Finally, the encapsulation of the counter ions X associated with a release of one equivalent of an acid accelerates the addition reaction (compare the blue and the green data in Fig. 1).

**Fig. 2:**
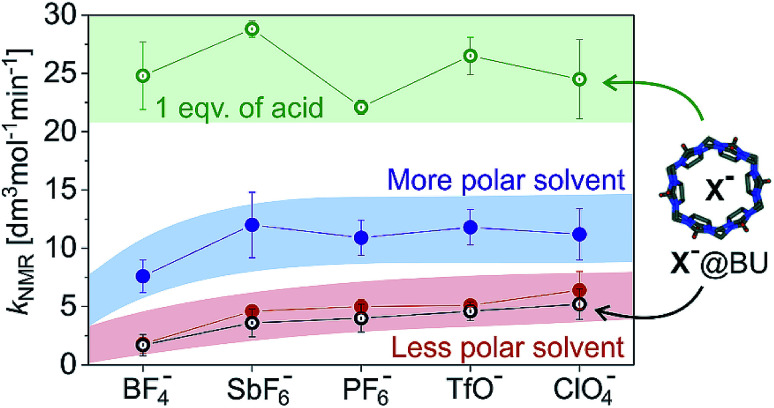
Total rates (*k*_NMR_ = *k*_1_ + *k*_2_) for methanol addition to 1-phenylpropyne catalysed by different (IPr)AuX salts (X^−^ is indicated on the *x*-axis). The experiments were performed in a mixture of methanol and dichloromethane: 1 : 2.4 (red, black) and 1 : 0.08 (blue, green), respectively. The hollow points refer to the experiment with addition of 1 equivalent of Bn-BU (black) or iPr-BU*H_2_SO_4_ (green) with respect to the concentration of the gold catalyst.

Fig. 2 summarizes the above findings for all investigated catalysts [(IPr)AuX] and allows us to analyse the trends. In the comparison of the different [(IPr)AuX] catalysts, the reaction rates are similar. Within the error bars of the experiment, we could conclude that the reaction mediated by [(IPr)Au(BF_4_)] is slower than the remaining reactions (Fig. 1 and 2). The difference between the other salts is small and can be only observed in the less polar reaction mixture (1 : 2.4 mixture of methanol and dichloromethane). Transitioning to almost pure methanol (1 : 0.08 mixture of methanol and dichloromethane), the differences between the catalytic properties of the salts diminish (the blue panel in Fig. 2).

Next, we tested the addition of 1 equivalent of iPr-BU*H_2_SO_4_ to the reaction mixture (equivalents of bambusurils relate to the concentration of the gold catalysts throughout this work). Addition of iPr-BU*H_2_SO_4_ leads to exchange of the anions and to release of the acid. Consequently, the reaction accelerates and the overall rate roughly doubles. Most importantly, the reaction catalysed by [(IPr)Au(BF_4_)] is not slower any more, but proceeds with more or less the same rate as the reactions catalysed by other gold salts. This result demonstrates that the anions got encapsulated and the presence of BF_4_^−^ no longer impairs the reaction.^29^ Instead, the reaction mixture contains the HSO_4_^−^ anions (in case of catalysts with X^−^ = BF_4_^−^, ClO_4_^−^, and PF_6_^−^) or a mixture of HSO_4_^−^ and X^−^ for X^−^ = TfO^−^ or SbF_6_^−^. Notably, the latter possibility seems to lead to slightly faster conversion (see Fig. 2).

Finally, we have evaluated the effect of adding 1 equivalent of Bn-BU. The reaction rates of all investigated reaction variants dropped by about 10–20%. The effect is small, but consistent for all experiments. The small drop but the same trend of the rate constants is inconsistent with the rates being affected by the active role of anions in the reaction path leading through the monoaurated intermediates. Instead, the effect is most probably connected with the p*K*_a_ of the conjugated acids in the given reaction mixture. The p*K*_a_ of the conjugated acid probably slightly dropped upon anion encapsulation.

Hence, we can conclude that the anions of the [(IPr)AuX] catalysts do not play an important role in methanol addition to an alkyne. The critical step of the deprotonation of the **A**H^+^ adduct to form the key monoaurated intermediate **A** can be mediated by solvent molecules or anions, but it is not the rate determining step and it does not affect the overall observed reaction rate. We did observe a slight dependence of the overall rate of the reaction on the anions X^−^ present; however, this dependence did not change based on whether the anion was free in solution or encapsulated in bambusurils. Therefore, this dependence most probably relates to the acidity of the conjugated acid and thus to the effect of HX on the rate-determining protodeauration step.

### Reaction pathway proceeding through diaurated intermediate B (catalysis by (PPh_3_)Au^+^)

The reaction pathway proceeding *via* diaurated intermediates does not involve any step where a specific role of an anion would be expected. Nevertheless, we have tested the effect of anion encapsulation, because this pathway allows us to interconnect the overall reaction rate with the degradation of the diaurated intermediates (*i.e.*, with the protodeauration rate). Therefore, we can directly asses the effect of the added acid on the protodeauration step and thus on the overall reaction rate. For comparison, we have tested also the effect of pure acid addition (*para*-toluenesulfonic acid, TsOH).

**Fig. 3:**
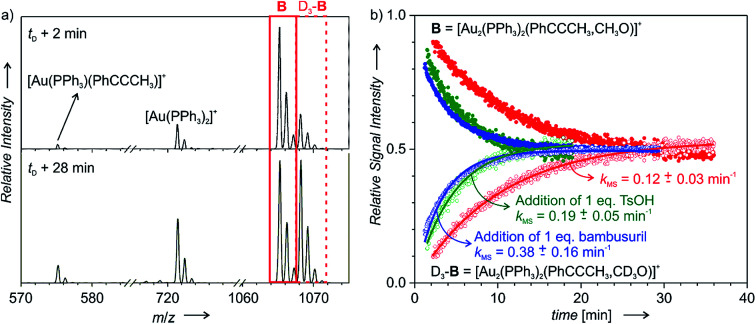
Delayed reactant labelling: (a) ESI-MS spectra recorded 2 and 28 min after adding CD_3_OH (time delay: 5 min) to the reaction mixture of PhCCCH_3_ with 1.25 mol% [Au(PPh_3_)Cl]/1.5 mol% AgSbF_6_/1.25 mol% bambusuril iPr-BU*H_2_SO_4_ in CH_3_OH. (b) Mutual time progression of integrated MS peak intensities of **B** and D_3_-**B** in the reaction mixture of PhCCCH_3_ with 1.25 mol% [Au(PPh_3_)SbF_6_] (in red). In addition, the reaction mixture contained 1.25 mol% iPr-BU*H_2_SO_4_ (blue) or 1.25 mol% TsOH (green). The solid lines are exponential fits (see eqn (S8–S11)†).

The diaurated intermediates [(PPh_3_)_2_Au_2_(PhCCCH_3_,CH_3_O)]^+^ (**B**) can be readily detected by electrospray ionization mass spectrometry (ESI-MS) and their lifetime can be determined by delayed reactant labelling (Fig. 3). This method consists of ESI-MS monitoring of intermediates with different isotopic labelling. We have used labelling with CH_3_OH and CD_3_OH reactants (we have shown previously that CD_3_OH does not affect the rate of the addition by a kinetic isotope effect). One of the isotopic labels (here CD_3_OH) is added to the reaction mixture with a time-delay. Right after this addition, the concentrations of CH_3_OH and CD_3_OH are in a 1 : 1 ratio. However, the signals of the detected intermediates [(PPh_3_)_2_Au_2_(PhCCCH_3_,CH_3_O)]^+^ (**B**) and [(PPh_3_)_2_Au_2_(PhCCCH_3_,CD_3_O)]^+^ (D_3_-**B**) are not in a 1 : 1 ratio, therefore their concentrations are not in equilibrium (Fig. 3a). Consequently, mutual time-evolution of the signals of **B** and D_3_-**B** reflects the establishing of equilibrium (Fig. 3b). Under the steady-state approximation, the exponential fit of this mutual evolution of the isotopic signals provides the rate of degradation of the corresponding intermediates in solution (for details see the ESI† and ref. 20). Here, the fitted rate corresponds to the rate of protodeauration of diaurated intermediates **B**.

Fig. 3b shows the kinetics of degradation of diaurated intermediates **B** in a plain reaction mixture (red) and with addition of 1 equiv. of *p*-toluenesulfonic acid (TsOH, green) or of 1 equiv. of iPr-BU*H_2_SO_4_ (blue). At the first sight, we can conclude that addition of TsOH as well as addition of iPr-BU*H_2_SO_4_ substantially accelerated protodeauration of the diaurated intermediates. The averages of multiple measurements under different conditions are summarized in Table 2.

**Table 2:** Overall rate constants *k*_NMR_^a^ for the addition of methanol to 1-phenylpropyne catalysed by [(PPh_3_)AuX] gold(i) determined from NMR experiments and rate constants *k*_MS_^b^ of degradation of diaurated intermediate **B** ([Au_2_(PPh_3_)_2_(PhCCCH_3_,CH_3_O)]^+^) determined by ESI-MS experiments

X	TsOH^c^ equiv.^e^	iPr-BU*H_2_SO_4_^d^ equiv.^e^	*k*_NMR_ [dm^3^ mol^−1^ min^−1^]	*k*_MS_ [min^−1^]
SbF_6_	—	—	0.12	0.12 ± 0.03
SbF_6_	0.5	—	—	0.20 ± 0.06
SbF_6_	1	—	0.39	0.19 ± 0.05
SbF_6_	2	—	0.62	0.30 ± 0.02
SbF_6_	4	—	0.80	0.48 ± 0.09
SbF_6_	—	0.5	0.28	0.33 ± 0.13
SbF_6_	—	1	0.40	0.38 ± 0.16
SbF_6_	—	2	0.40	0.42 ± 0.10
SbF_6_	—	4	0.39	—
OTf	—	—	0.19	0.13 ± 0.02
PF_6_	—	—	0.16	0.14 ± 0.04
OTf	—	1	0.30	0.25 ± 0.03
PF_6_	—	1	0.26	0.26 ± 0.09

a The reaction was performed in CD_3_OD (0.6 M 1-phenylpropyne with 1.25 mol% [Au(PPh_3_)X]). The rate constants were determined by the kinetic modelling of the decay of the reactant monitored by NMR.

b The reaction was performed in CH_3_OH (0.06 M 1-phenylpropyne with 1.25 mol% [Au(PPh_3_)X]) diluted after the time delay 5 min with CD_3_OH. The rate constants were determined by delayed reactant labelling.

c Different mol% of *p*-toluenesulfonic acid (TsOH) was added to the reaction mixture.

d Different mol% of bambusuril iPr-BU*H_2_SO_4_ was added to the reaction mixture.

e Equivalents refer to the concentration of [(PPh_3_)Au]^+^. 1 equivalent is 1.25 mol% concentration.

**Fig. 4:**
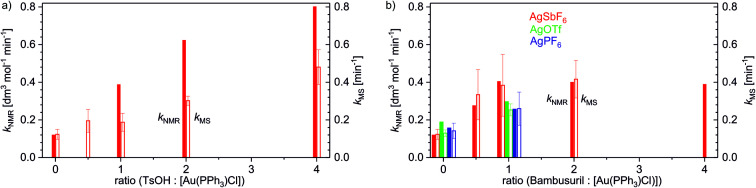
The effect of (a) TsOH and (b) bambusuril iPr-BU*H_2_SO_4_ on the rate constants of degradation *k*_MS_ of the intermediate **B** ([(PPh_3_)_2_Au_2_(PhCCCH_3_,CH_3_O)]^+^, *m*/*z* 1065) determined by ESI-MS experiments and on the overall rate constants *k*_NMR_ for the addition of methanol to 1-phenylpropyne catalysed by [(PPh_3_)AuX] (X = SbF_6_, PF_6_, OTf) determined by the kinetic modelling of the decay of the reactant monitored by NMR.

Using the delayed reactant labelling method and monitoring the overall reaction rate of the addition reaction with NMR, we compared the effects of different reaction conditions (Table 2). Firstly, we looked deeper into the effect of the added acid on methanol addition to 1-phenylpropyne catalysed by [(PPh_3_)Au(SbF_6_)]. The NMR experiments showed that with addition of 1 equivalent of TsOH (equivalents refer to the concentration of the gold salt), the overall rate triples (see Fig. 4). With more equivalents of TsOH, the rate still increases but with decreasing slope.

Protodeauration is the rate-determining step for the addition reaction, therefore we should observe similar kinetic effects for the rates determined from ESI-MS experiments. In agreement, the rate of protodeauration increases with increasing concentration of the acid. The slope of the increase is somewhat smaller than the slope determined for the overall rate. However, it must be emphasized that the reaction mixture was 10 times diluted in the experiments monitored by ESI-MS. Hence, we can conclude that addition of the acid increases the rate of the addition reaction and this effect is mirrored in the rate of protodeauration of individual intermediates.

Next, we tested the effect of the bambusuril complexed with one equivalent of an acid, iPr-BU*H_2_SO_4_, on methanol addition to 1-phenylpropyne catalysed by [(PPh_3_)Au(SbF_6_)]. As expected, adding of 1 equivalent of iPr-BU*H_2_SO_4_ led to acceleration of the overall reaction (*k*_NMR_) in an almost identical way to adding 1 equivalent of TsOH. However, unlike with TsOH further addition of iPr-BU*H_2_SO_4_ did not increase the rate of the reaction any more. Hence, iPr-BU*H_2_SO_4_ does not behave as an acid *per se*. The small acidity of the H[HSO_4_@iPr-BU] complex is in agreement with the suggested acidity drop of the conjugated acids HX upon anion encapsulation (see the previous chapter).

The NMR kinetics suggested that we have a fast exchange of SbF_6_^−^ and HSO_4_^−^ in the cavity of the bambusuril (Fig. S20 and S21†). This fast exchange may affect the acidity of the protonated bambusurils and it seems to be connected with a release of one equivalent of protons to the solution. In addition, the free HSO_4_^−^ anion will be in equilibrium with H^+^ and SO_4_^2−^ further increasing the acidity of the solution. Adding more than 1 equivalent of iPr-BU*H_2_SO_4_ does not further increase the concentration of protons in solution, because more than 1 equivalent of iPr-BU*H_2_SO_4_ cannot be involved in the fast exchange.

In parallel, we have again tested in analogous experiments the effect of adding iPr-BU*H_2_SO_4_ on the protodeauration rate (*k*_MS_). In complete agreement, we detected acceleration of protodeauration of the intermediates when adding up to 1 equivalent of the bambusuril complex. Further addition of iPr-BU*H_2_SO_4_ did not affect the intermediates any more (Fig. 4b). Remarkably, the acceleration of protodeauration was slightly larger when adding 1 equiv. iPr-BU*H_2_SO_4_ than when adding 1 equiv. TsOH (compare hollow bars in Fig. 4a and b).

Lastly, we tested the effect of different counter ions in the catalyst (X = SbF_6_, PF_6_, TfO) and the effect of adding iPr-BU*H_2_SO_4_. In the absence of bambusuril, all tested catalysts mediated the addition reaction with the same rate (*k*_NMR_) within the error of the experiment. Adding 1 equivalent of iPr-BU*H_2_SO_4_ led to the expected acceleration of the reaction. The reaction catalysed by [(PPh_3_)Au(SbF_6_)] was accelerated more than those catalysed by [(PPh_3_)Au(PF_6_)] (PF_6_^−^ displaces HSO_4_^−^ in the cavity of iPr-BU) and by [(PPh_3_)Au(TfO)] (TfO^−^ exchanges with HSO_4_^−^ in the cavity). We have reproduced the very same effect also for the protodeauration rate (*k*_MS_) of the diaurated intermediates (see Fig. 4b).

The different effects of anions detected after their encapsulation in iPr-BU must be connected with the different acidity of the H[X@iPr-BU] acids. As demonstrated above, the original complex iPr-BU*H_2_SO_4_ (that is H[HSO_4_@iPr-BU]) does not behave itself as an acid in the methanol solution. Once, the HSO_4_^−^ is released to the solution, the proton also releases to the solution. However, the acidity is affected by the p*K*_a_ of H[X@iPr-BU] in methanol, which can differ for different anions. The released HSO_4_^−^ can also contribute to the overall acidity, but probably to a lesser extent. The larger acidity of H[SbF_6_@iPr-BU] as compared to H[PF_6_@iPr-BU] and to H[TfO@iPr-BU] is also consistent with results obtained for the [(IPr)AuX] catalysis (Fig. 2).

## Conclusions

We presented a new mechanistic tool for probing the effects of anions in reaction mechanisms. Bambusuril macrocycles bind anions with large binding constants and thus effectively encapsulate them within their cavity. Under the conditions that bambusurils do not participate in any reaction themselves, they might be an ideal probe to demonstrate the active role of anions in given reactions.

We have used bambusurils to probe the effect of anions on gold(i) mediated addition of methanol to 1-phenylpropyne. We show that the rates of the reactions catalysed by [(IPr)AuX] proceeding through neutral monoaurated intermediates slightly depend on the anion X. However, this dependence is not connected with the reactivity of the free anions in solution, because the dependence is almost unaffected by encapsulation of the anions into the cavity of bambusurils. Therefore, most probably, the reaction is affected by the p*K*_a_ of the conjugated acid HX and thus by the effect of HX on the rate-determining step of the reaction – protodeauration of the intermediates.

We have also probed the anion effect in the reaction proceeding through the diaurated reaction intermediates (*i.e.*, [(PPh_3_)AuX] catalysed methanol addition to 1-phenylpropyne). The results showed a small anion effect, again most probably connected with the p*K*_a_ of the conjugated acids HX. For this reaction pathway, we could compare the overall reaction rates determined by NMR with the rates of protodeauration of diaurated intermediates determined by electrospray ionization mass spectrometry. The rate dependencies are consistent. The overall rate constants are affected in the very same way as the rates of protodeauration. This demonstrates that the nature of anions X^−^ affects the rate-determining protodeauration step that depends on the acidity of the reaction mixture and hence on the p*K*_a_ of the conjugated acids HX.

This result does not confirm previously published reports on the key effect of anions for deprotonation of the initial adduct between gold-activated alkyne and an alcohol at least for the system under study. The results might differ for different alkynes and nucleophiles and might be strongly affected by solvent.^30^ For example, if the reaction were to be run under conditions that meant that protodeauration was not the rate determining step and if the solvent molecules were incapable of mediating proton shuttling, the role of anions might gain importance.

The mechanistic probe with using bambusurils is a simple and straightforward way to prove the potential anion effect and should be used rather than relying only on theoretical modelling. Theoretical modelling usually cannot take all details of the reaction mixture into account. The expected reaction mechanism based on knowledge and intuition usually steers the theoretical calculations to the foreseen outcome. Often, this gives the correct rationale, but sometimes, unexpected reaction pathways might be overlooked.

## Conflicts of interest

There are no conflicts to declare.

## Supplementary Material

FD-220-C9FD00038K-s001
