# Supplementary material for: Bambusurils as a mechanistic tool for probing anion effects
Source: Faraday Discuss. 2019 Jun 4;220:58–70. doi: 10.1039/c9fd00038k (PMC8609304; doi:10.1039/c9fd00038k)
Supplement: FD-220-C9FD00038K-s001 [file FD-220-C9FD00038K-s001.pdf]

Electronic Supplementary Material (ESI) for Faraday Discussions.
This journal is © The Royal Society of Chemistry 2019

SUPPORTING INFORMATION

Bambusurils as a mechanistic tool to probe the anions effect in reactions

Lucie Jašíková,[a] Maitê Rodrigues,[a] Jana Lapešová,[b] Tomáš Lízal,[b] Vladimír Šindelář[b] and Jana Roithová*[c]

Content

Preparation of reaction mixtures for NMR spectroscopy experiments S2
(Tables S1 – S3)
Results of NMR spectroscopy kinetic experiments S5
(Scheme S1 and S2, Equations S1 – S7, Tables S4 and S5, Figures S1 and S2)
NMR spectra S10
(Figures S3 – S13)
Preparation of reaction mixtures for ESI-MS experiments S21
(Tables S5 – S7)
Delayed reactant labelling method S24
(Scheme S3, Equations S8 – S11)
Results of ESI-MS kinetic experiments S26
(Table S8)
Synthetic protocols and characterization of bambusurils S28
(NMR spectra: Figures S14 – S19)
NMR Competition Experiments S33
(Figures S21 and S22)

Preparation of reaction mixtures for NMR spectroscopy experiments

I) Experiments with [(IPr)AuX] and iPr-BU*H_2SO_4

The stock solution **A** was prepared by mixing of AgX (Table S1) dissolved in 0.6 ml of CD_3OD and 15.4 mg of AuCl(IPr) dissolved in 0.2 ml CD_2Cl_2. The reaction mixture was mixed for 30 minutes and filtered through a PTFE filter (pore size 0.2 µm) to remove precipitated AgCl.

Final reaction mixtures without iPr-BU*H_2SO_4 were prepared by mixing of 200 µl of the stock solution **A**, 62 µl of $PhCCCH_3$, 53 µl of toluene, and 516 µl of CD_3OD. Reaction mixture was immediately monitored by NMR spectroscopy.

Final reaction mixtures with iPr-BU*H_2SO_4 were prepared by mixing of 8.9 mg of iPr-BU*H_2SO_4, 200 µl of the stock solution **A**, 62 µl of $PhCCCH_3$, 53 µl of toluene, and 516 µl of CD_3OD. Reaction mixture was immediately monitored by NMR spectroscopy.

Table S1. Using of different silver salts

	[mg]
$AgBF_4$	5.8
$AgSbF_6$	10.2
AgOTf	7.6
$AgPF_6$	7.5
$AgClO_4$	6.2

II) Experiments with [(IPr)AuX] Bn-BU

The stock solution **A** was prepared by mixing of AgX (Table S1) dissolved in 0.2 ml of CD_3OD and 15.4 mg of AuCl(IPr) dissolved in 0.6 ml CD_2Cl_2. The reaction mixture was mixed for 30 minutes and filtered through a PTFE filter (pore size 0.2 µm) to remove precipitated AgCl.

Final reaction mixtures without Bn-BU were prepared by mixing of 200 μl of the stock solution **A**, 62 μl of $PhCCCH_3$, 53 μl of toluene, 350 μl of CD_2Cl_2, and 166 μl of CD_3OD. Reaction mixture was immediately monitored by NMR spectroscopy.

Final reaction mixtures with Bn-BU were prepared by mixing of 12.5 mg of Bn-BU, 200 μl of the stock solution **A**, 62 μl of $PhCCCH_3$, 53 μl of toluene, 350 μl of CD_2Cl_2, and 166 μl of CD_3OD. Reaction mixture was immediately monitored by NMR spectroscopy.

III) Experiments with [(PPh_3)Au(SbF_6)] and different concentration of *p*-toluenesulfonic acid (TsOH)

The stock solution **A** was prepared by mixing of 10.2 mg of $AgSbF_6$ dissolved in 0.6 ml of CD_3OD and 12.3 mg of AuCl(PPh_3) dissolved in 0.2 ml CD_2Cl_2. The reaction mixture was mixed for 30 minutes and filtered through a PTFE filter (pore size 0.2 μm) to remove precipitated AgCl.

Final reaction mixtures were prepared by mixing of 200 μl of the stock solution **A**, 100 μl of the stock solution of TsOH (for the composition of the solution see Table S2), 62 μl of $PhCCCH_3$, 53 μl of toluene, and 416 μl of CD_3OD. Reaction mixture was immediately monitored by NMR spectroscopy.

Table S2. Composition of the solution of TsOH

TsOH [mol%]	1.25	2.50	5.00
TsOH [mg]	9.5	19.0	38.0
CD_3OD [μl]	200	200	200

IV) Experiments with [(PPh_3)Au(SbF_6)] and iPr-BU*H_2SO_4

The stock solution **A** was prepared by mixing of 10.2 mg of $AgSbF_6$ dissolved in 0.6 ml of CD_3OD and 12.3 mg of AuCl(PPh_3) dissolved in 0.2 ml CD_2Cl_2. The reaction mixture was mixed for 30 minutes and filtered through a PTFE filter (pore size 0.2 μm) to remove precipitated AgCl.

Final reaction mixtures were prepared by mixing of 138 µl of the stock solution **A**, 69 µl of the stock solution of iPr-BU*H_2SO_4 (for the composition of the solution see Table S3), 43 µl of $PhCCCH_3$, 36 µl of toluene, and 287 µl of CD_3OD. Reaction mixture was immediately monitored by NMR spectroscopy.

Table S3. Composition of the solution of iPr-BU*H_2SO_4

**1** [mol%]	0.63	1.25	2.50	5.00
**1** [mg]	3.1	6.1	12.2	24.4
CD_3OD [µl]	69	69	69	69

Results of NMR spectroscopy experiments

The NMR experiments were recorded using a Varian NMR System (300 MHz) and the δ scale was referenced to the solvent residual peak at δ = 3.31 ppm. We used toluene as an internal standard. The solutions of a catalyst and reactants were mixed and immediately probed by the NMR instrument.

We have fitted the time-dependence of the intensities of reactant and products using rate equations based on Scheme S1. For reactions catalyzed by (IPr)Au^+, we have evaluated all rate constants (k_1, k_2, k_{11}, k_{22}). The reactions catalyzed by $(PPh_3)Au^+$ are slower, therefore we have evaluated only the rate of concentration decrease of the alkyne.

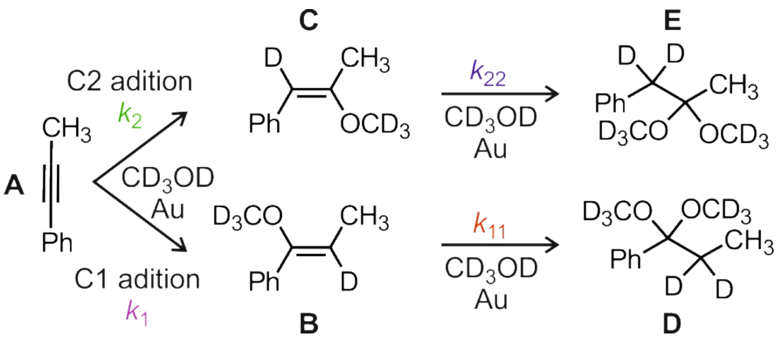

Scheme S1. Addition of methanol on 1-phenylpropyne.

Kinetic model

1) (IPr)Au^+ catalysed reaction

Based on Scheme S1 following differential equation was suggested:

$$\frac{d[\mathrm{A}]}{dt} = -(k_1 + k_2)[Au][\mathrm{A}] = -(k_{1f} + k_{2f})[\mathrm{A}] \quad \text{(S1)}$$

$$\frac{d[\mathrm{B}]}{dt} = k_1[Au][\mathrm{A}] - k_{11}[Au][\mathrm{B}] = k_{1f}[\mathrm{A}] - k_{11f}[B] \quad \text{(S2)}$$

$$\frac{d[\mathrm{C}]}{dt} = k_2[Au][\mathrm{A}] - k_{22}[Au][\mathrm{C}] = k_{2f}[\mathrm{A}] - k_{22f}[C] \quad \text{(S3)}$$

$$\frac{d[\mathrm{D}]}{dt} = k_{11}[Au][\mathrm{B}] = k_{11f}[B] \quad \text{(S4)}$$

$$\frac{d[\mathrm{E}]}{dt} = k_{22}[Au][\mathrm{C}] = k_{22f}[C] \quad \text{(S5)}$$

These equations were used as a kinetic model for the fitting of experimental data with mathematic software GNU Octave. Rate constants were free fit parameters and equations were solved numerically. We used the least squares method for the fitting of experimental data. The final rate constants $k_{1,2,11,22}$ (Table S2) were given by equation S6, where [Au] = 0.0075 mol.dm^{-3}.

$$k_{1,2,11,22} = \frac{k_{1f,2f,11f,22f}}{[Au]} \quad \text{(S6)}$$

2) $(PPh_3)Au^+$ catalysed reaction

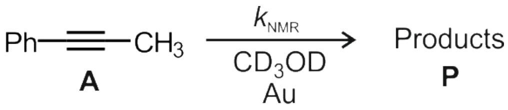

Scheme S2. Addition of methanol on 1-phenylpropyne.

Based on Scheme S2 following differential equation was suggested:

$$\frac{d[\mathrm{A}]}{dt} = - k_{NMR}[\mathrm{A}] \quad \text{(S7)}$$

This equation was used as a kinetic model for the fitting of experimental data with mathematic software GNU Octave. Rate constant k_{NMR} was free fit parameter and equation was solved numerically. We used the least squares method for the fitting of experimental data. Obtained rate constants are shown in Table S5.

Results of NMR experiments

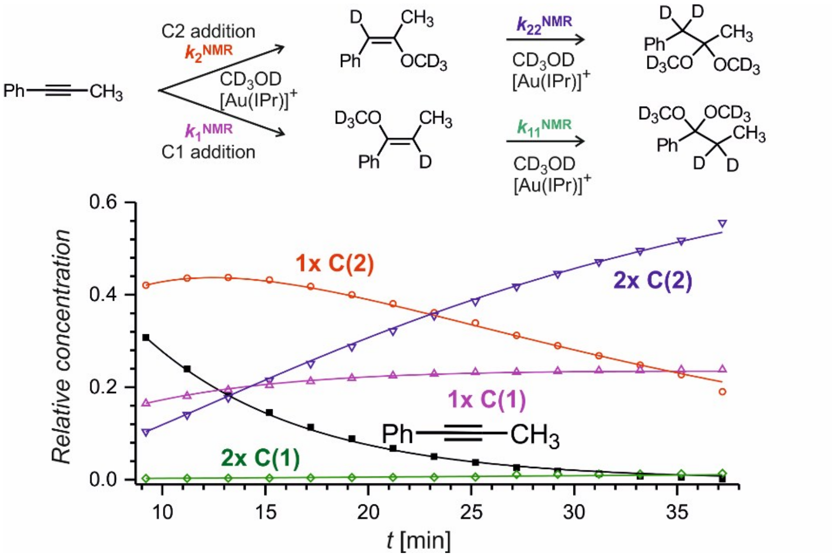

Figure S1_part 1: Explanation of the measured data. The kinetic data in the figures below show relative concentrations of 1-phenylpropyne (the concentration decreases with time) and concentrations of the products of methanol addition to the C(1) and C(2) carbon atoms of 1-phenylpropyne. We will not color-code the individual channels, because we color-code addition of bambusurils. However, the rate constants for all channels describing the fitted curves are summarized in Table 1.

Figure S1_part 2 (the next page**)**. Relative ratios of 1-phenylpropyne, the products of C1 and C2 additions as a function of the reaction time as monitored by NMR spectroscopy for 1.25 mol% [AuCl(IPr)]/1.5 mol% a,b) $AgSbF_6$, c,d) $AgPF_6$, e,f) $AgClO_4$. The solid lines correspond to fits obtained by the *Octave* program assuming the Scheme S1; the corresponding rate constants are listed in Table 1.

The effect of encapsulation of X in a bambusuril

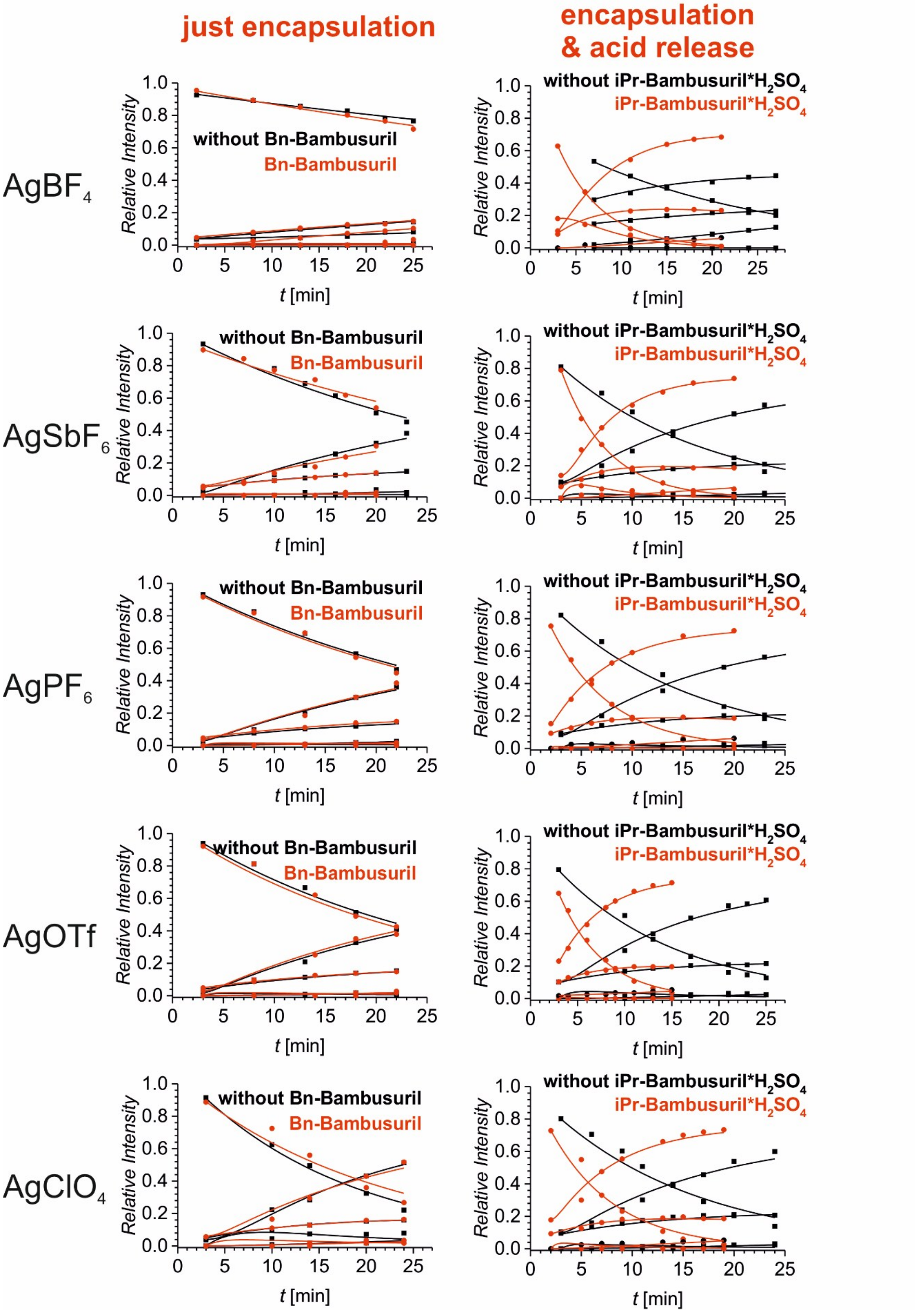

Table S4. Rate constants k_{NMR} obtained by NMR spectroscopy.[a]

X^-	c ($PhCCCH_3$) [$mol.dm^{-3}$]	$Au(PPh_3)Cl$ [mol%]	TsOH[b] [mol%]	iPr-BU*H_2SO_4[c] [mol%]	k_{NMR} [$dm^3.mol.^{-1}.min^{-1}$]
SbF_6	0.3	2.50	-	-	0.20
SbF_6	0.6	1.25	-	-	0.12
SbF_6	1.2	0.63	-	-	0.065
SbF_6	0.6	2.50	-	-	0.09
SbF_6	0.6	5.00	-	-	0.05
SbF_6	0.6	1.25	1.25	-	0.39
SbF_6	0.6	1.25	2.50	-	0.62
SbF_6	0.6	1.25	5.00	-	0.80
OTf	0.6	1.25	-	-	0.19
PF_6	0.6	1.25	-	-	0.16
OTf	0.6	1.25	-	1.25	0.30
PF_6	0.6	1.25	-	1.25	0.26
SbF_6	0.6	1.25	-	0.63	0.28
SbF_6	0.6	1.25	-	1.25	0.40
SbF_6	0.6	1.25	-	2.50	0.40
SbF_6	0.6	1.25	-	5.00	0.39

[a] CD_3OD was added to the reaction mixture of different concentration of 1-phenylpropyne with different mol% [$Au(PPh_3)Cl$]/AgX.

[b] Different mol% *p*-toluenesulfonic acid (TsOH) was added to the reaction mixture.

[c] Different mol% iPr-BU*H_2SO_4 was added to the reaction mixture.

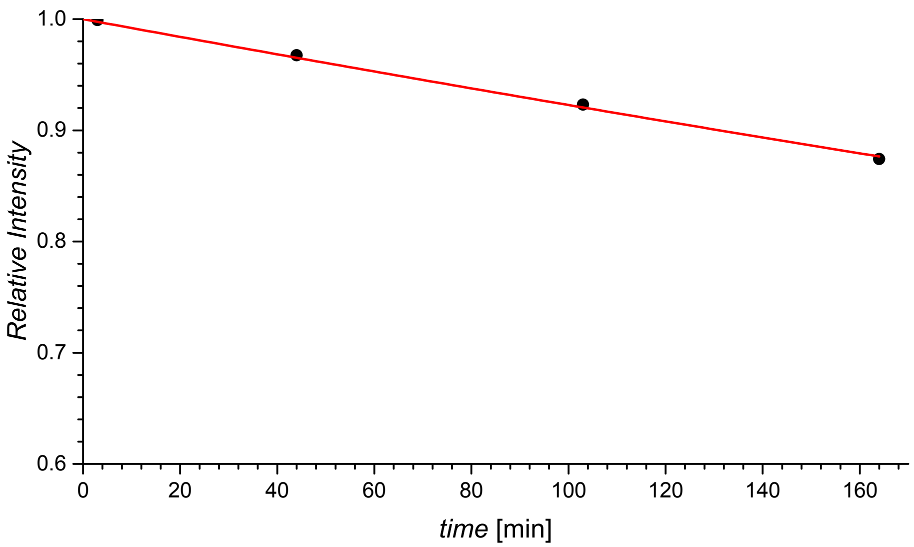

Figure S2. Relative ratio of 1-phenylpropyne. Red full curve represents fit obtained in GNU Octave.

NMR spectra

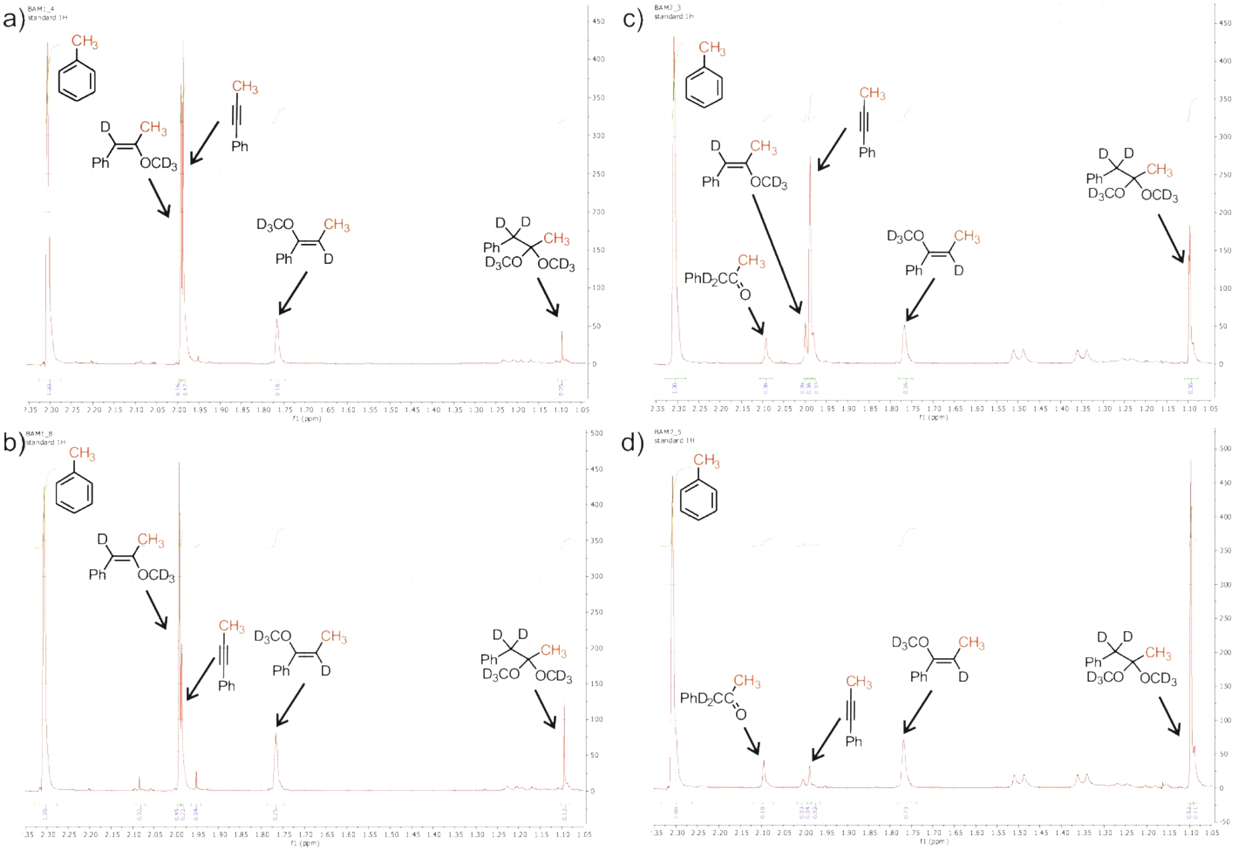

Figure S3. ^{1}H NMR spectra of the reaction mixture of 1.25 mol% [AuCl(IPr)]/1.5 mol% [$AgBF_4$] a) 10 and b) 27 minutes after the addition of the solution of catalyst to the solution of $PhCCCH_3$ and toluene in CD_3OD/CD_2Cl_2 (1:0.08). ^{1}H NMR spectra of the reaction mixture of 1.25 mol% [AuCl(IPr)]/1.5 mol% [$AgBF_4$]/ 1.25 mol% iPr-bambusuril **1** c) 5 and d) 10 minutes after the addition of the solution of catalyst to the solution of $PhCCCH_3$ and toluene in CD_3OD/CD_2Cl_2 (1:0.08). The signals correspond to the methyl groups shown in red in the corresponding formulas.

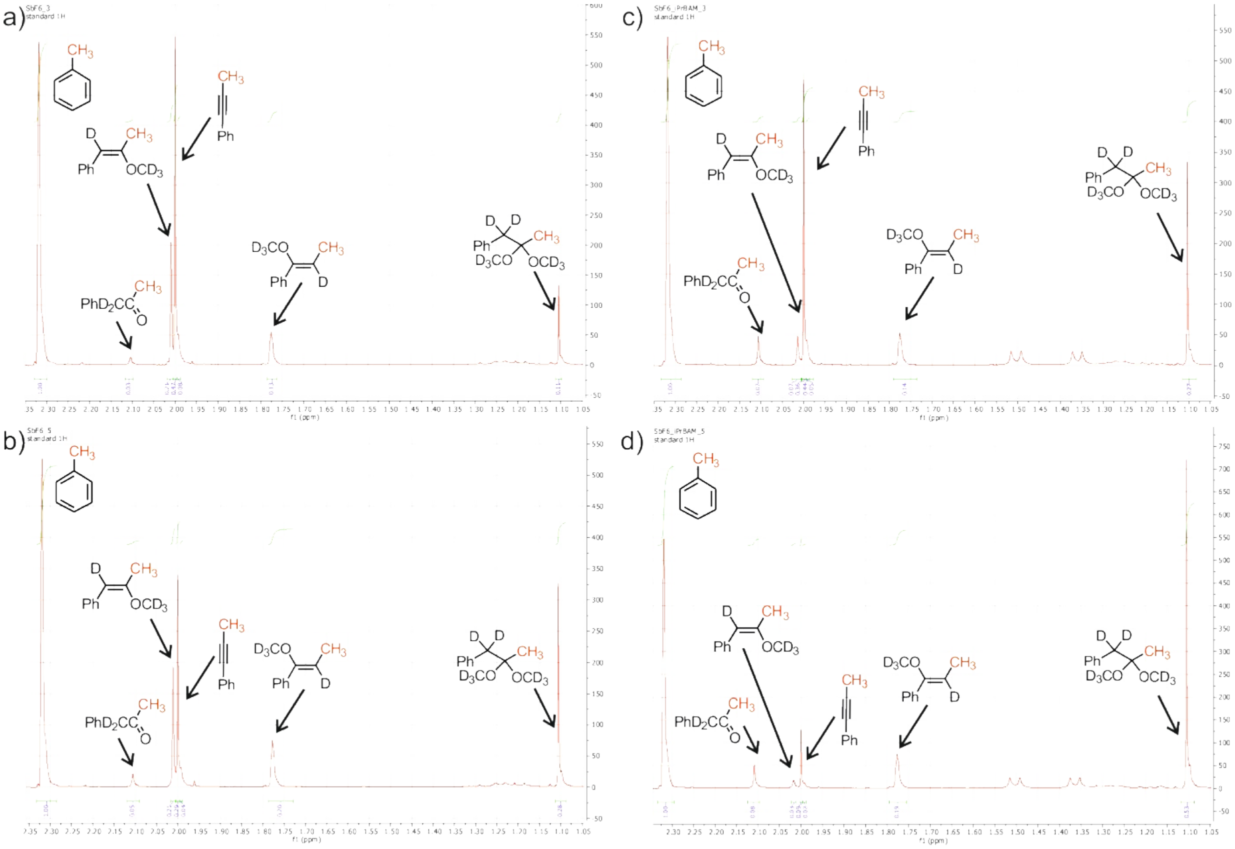

Figure S4. ^{1}H NMR spectra of the reaction mixture of 1.25 mol% [AuCl(IPr)]/1.5 mol% [$AgSbF_6$] a) 6 and b) 11 minutes after the addition of the solution of catalyst to the solution of $PhCCCH_3$ and toluene in CD_3OD/CD_2Cl_2 (1:0.08). ^{1}H NMR spectra of the reaction mixture of 1.25 mol% [AuCl(IPr)]/1.5 mol% [$AgSbF_6$]/ 1.25 mol% iPr-bambusuril **1** c) 6 and d) 12 minutes after the addition of the solution of catalyst to the solution of $PhCCCH_3$ and toluene in CD_3OD/CD_2Cl_2 (1:0.08). The signals correspond to the methyl groups shown in red in the corresponding formulas.

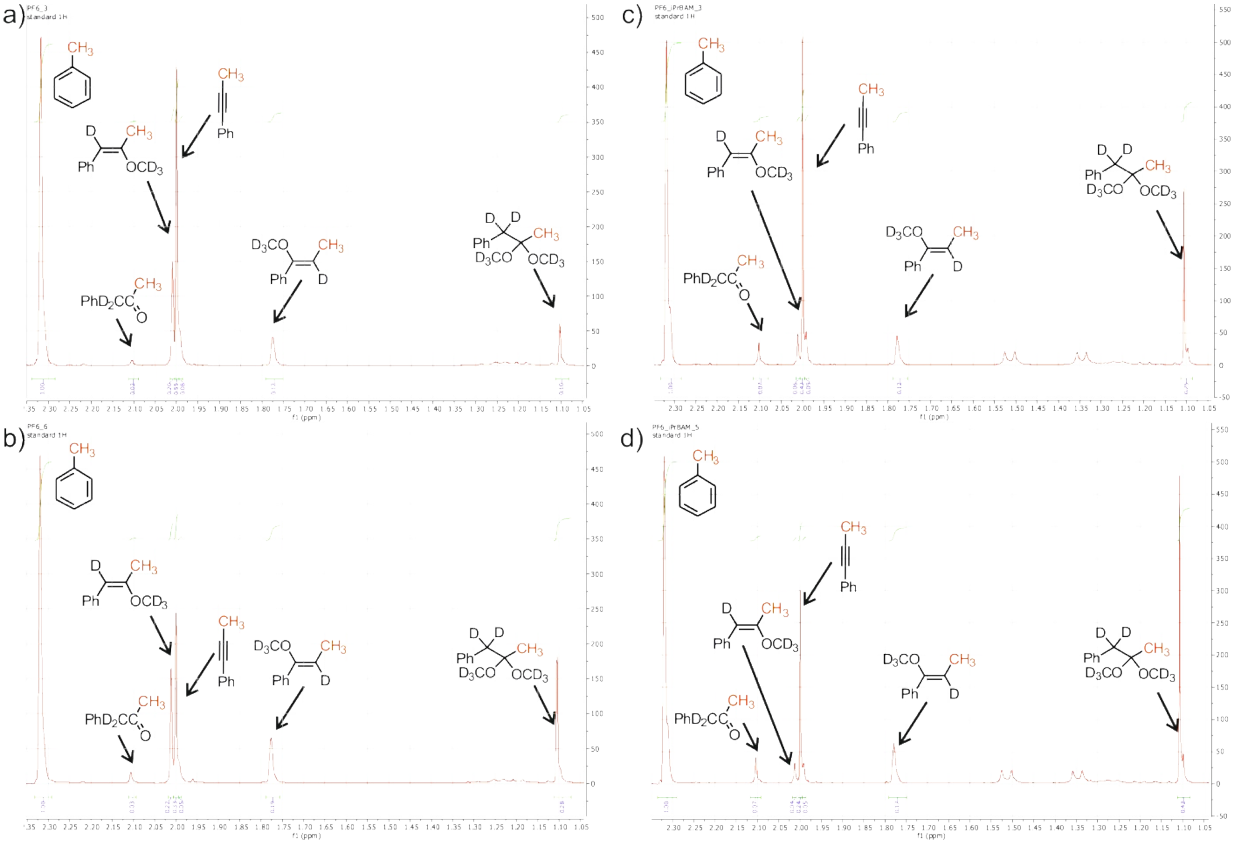

Figure S5. ^{1}H NMR spectra of the reaction mixture of 1.25 mol% [AuCl(IPr)]/1.5 mol% [$AgPF_6$] a) 5 and b) 11 minutes after the addition of the solution of catalyst to the solution of $PhCCCH_3$ and toluene in CD_3OD/CD_2Cl_2 (1:0.08). ^{1}H NMR spectra of the reaction mixture of 1.25 mol% [AuCl(IPr)]/1.5 mol% [$AgPF_6$]/ 1.25 mol% iPr-bambusuril **1** c) 6 and d) 9 minutes after the addition of the solution of catalyst to the solution of $PhCCCH_3$ and toluene in CD_3OD/CD_2Cl_2 (1:0.08). The signals correspond to the methyl groups shown in red in the corresponding formulas.

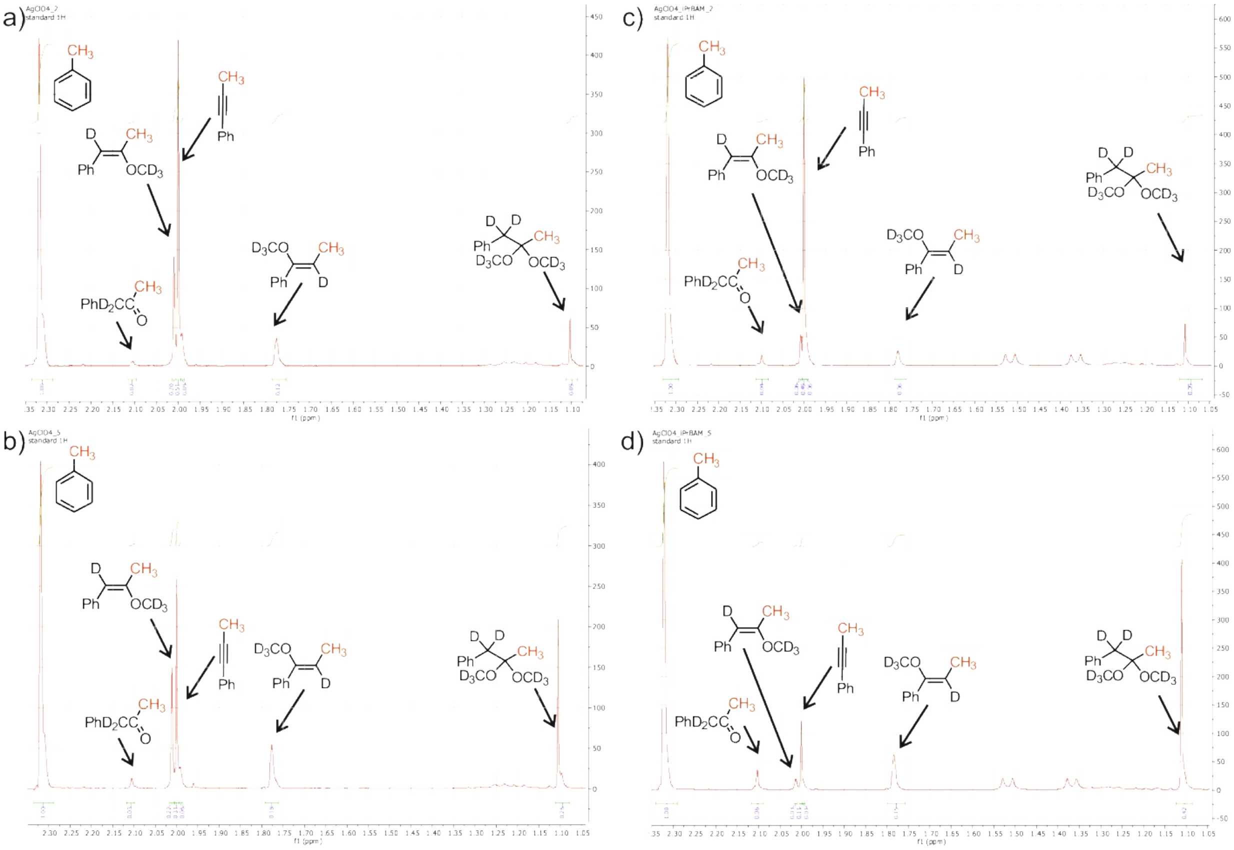

Figure S6. ^{1}H NMR spectra of the reaction mixture of 1.25 mol% [AuCl(IPr)]/1.5 mol% [$AgClO_4$] a) 4 and b) 9 minutes after the addition of the solution of catalyst to the solution of $PhCCCH_3$ and toluene in CD_3OD/CD_2Cl_2 (1:0.08). ^{1}H NMR spectra of the reaction mixture of 1.25 mol% [AuCl(IPr)]/1.5 mol% [$AgClO_4$]/ 1.25 mol% iPr-bambusuril **1** c) 3 and d) 10 minutes after the addition of the solution of catalyst to the solution of $PhCCCH_3$ and toluene in CD_3OD/CD_2Cl_2 (1:0.08). The signals correspond to the methyl groups shown in red in the corresponding formulas.

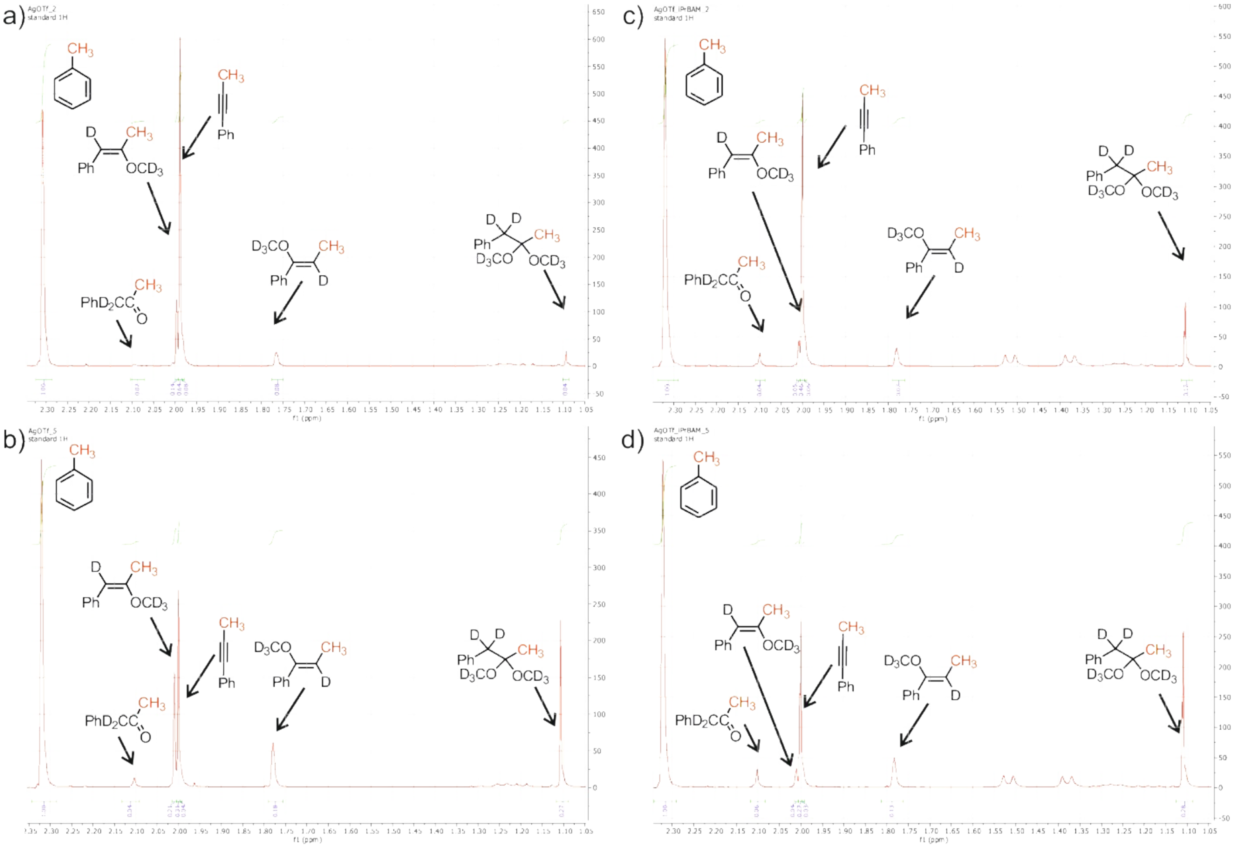

Figure S7. ^{1}H NMR spectra of the reaction mixture of 1.25 mol% [AuCl(IPr)]/1.5 mol% [AgOTf] a) 3 and b) 11 minutes after the addition of the solution of catalyst to the solution of $PhCCCH_3$ and toluene in CD_3OD/CD_2Cl_2 (1:0.08). ^{1}H NMR spectra of the reaction mixture of 1.25 mol% [AuCl(IPr)]/1.5 mol% [AgOTf]/ 1.25 mol% iPr-bambusuril **1** c) 4 and d) 7 minutes after the addition of the solution of catalyst to the solution of $PhCCCH_3$ and toluene in CD_3OD/CD_2Cl_2 (1:0.08). The signals correspond to the methyl groups shown in red in the corresponding formulas.

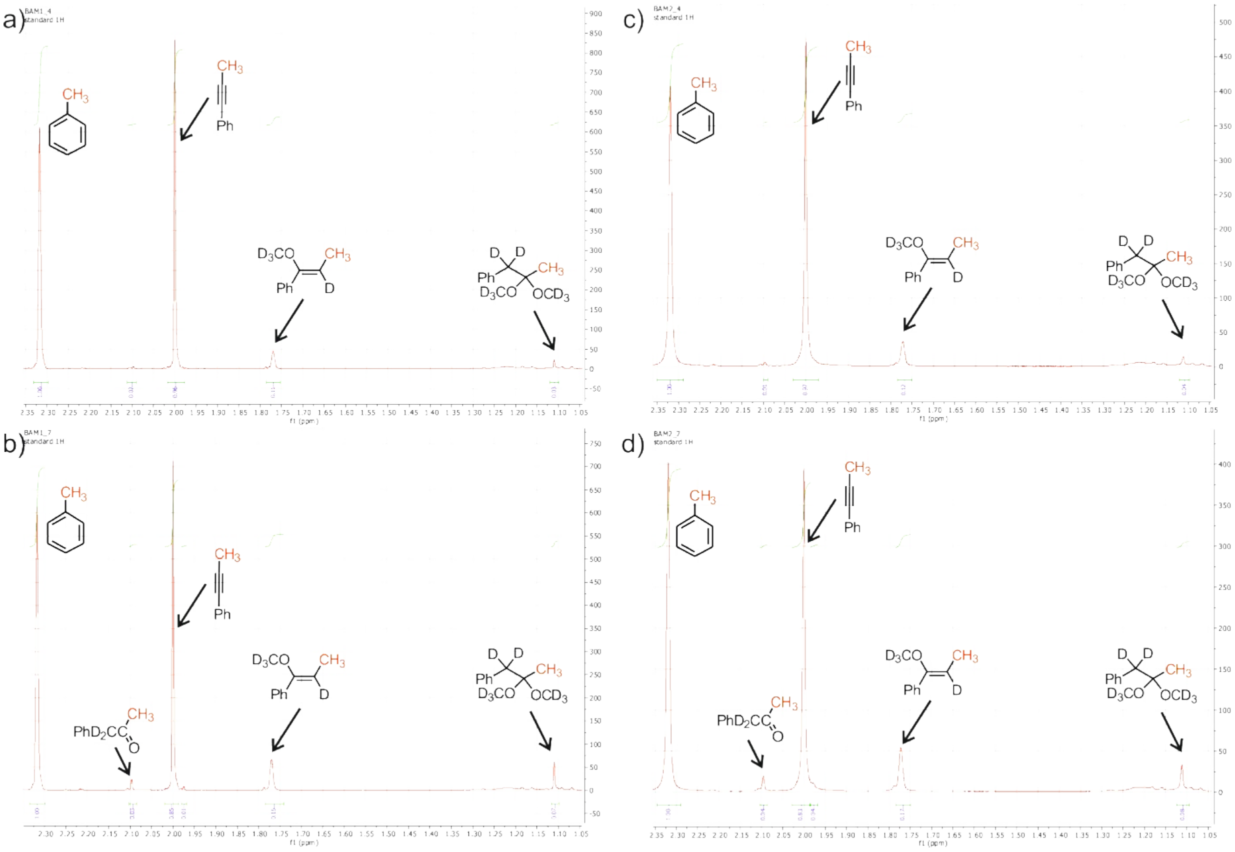

Figure S8. ^{1}H NMR spectra of the reaction mixture of 1.25 mol% [AuCl(IPr)]/1.5 mol% [$AgBF_4$] a) 13 and b) 25 minutes after the addition of the solution of catalyst to the solution of $PhCCCH_3$ and toluene in CD_3OD/CD_2Cl_2 (1:2.4). ^{1}H NMR spectra of the reaction mixture of 1.25 mol% [AuCl(IPr)]/1.5 mol% [$AgBF_4$]/ 1.25 mol% Bn-bambusuril **2** in CD_3OD/CD_2Cl_2 c) 13 and d) 25 minutes after the addition of the solution of catalyst to the solution of $PhCCCH_3$ and toluene in CD_3OD/CD_2Cl_2 (1:2.4). The signals correspond to the methyl groups shown in red in the corresponding formulas.

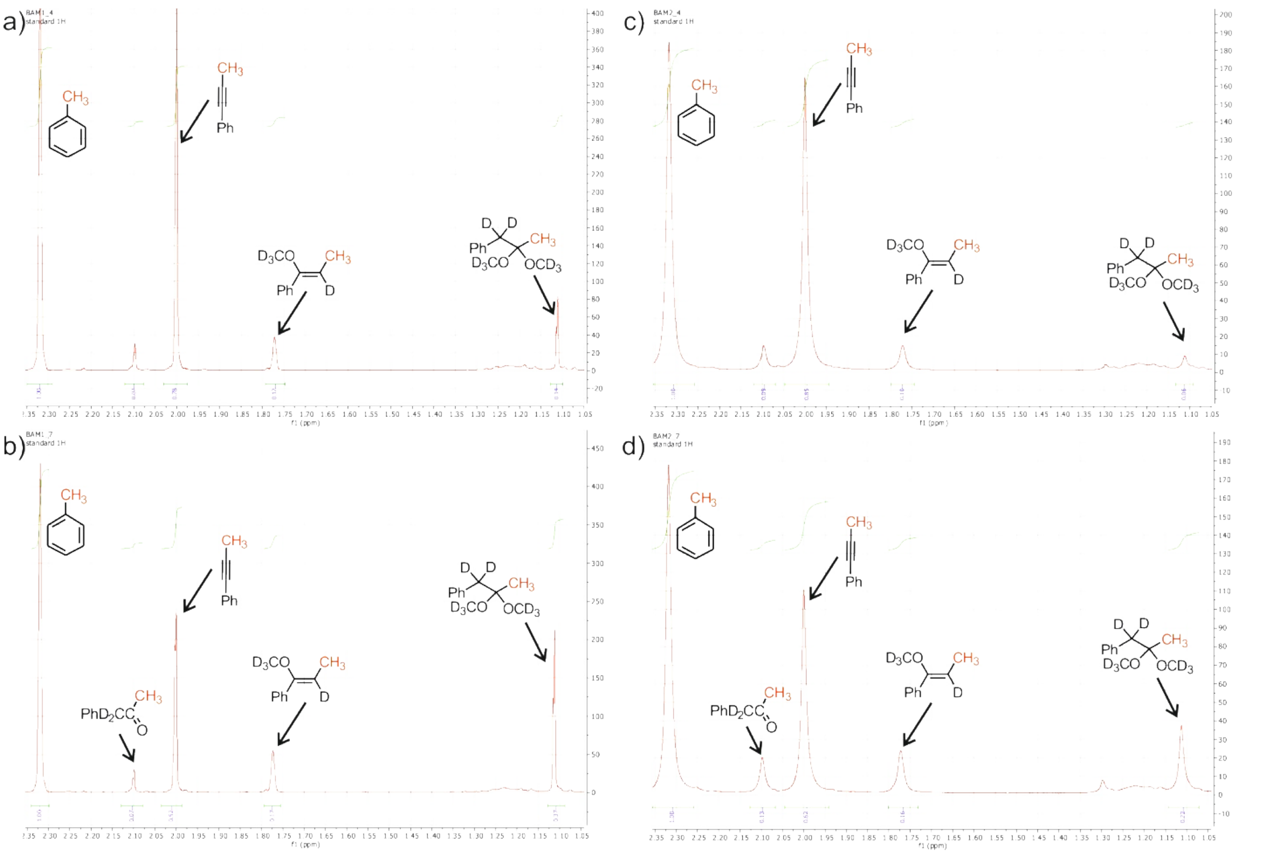

Figure S9. ^{1}H NMR spectra of the reaction mixture of 1.25 mol% [AuCl(IPr)]/1.5 mol% [$AgSbF_6$] a) 13 and b) 23 minutes after the addition of the solution of catalyst to the solution of $PhCCCH_3$ and toluene in CD_3OD/CD_2Cl_2 (1:2.4). ^{1}H NMR spectra of the reaction mixture of 1.25 mol% [AuCl(IPr)]/1.5 mol% [$AgSbF_6$]/ 1.25 mol% Bn-bambusuril **2** in CD_3OD/CD_2Cl_2 c) 10 and d) 20 minutes after the addition of the solution of catalyst to the solution of $PhCCCH_3$ and toluene in CD_3OD/CD_2Cl_2 (1:2.4). The signals correspond to the methyl groups shown in red in the corresponding formulas.

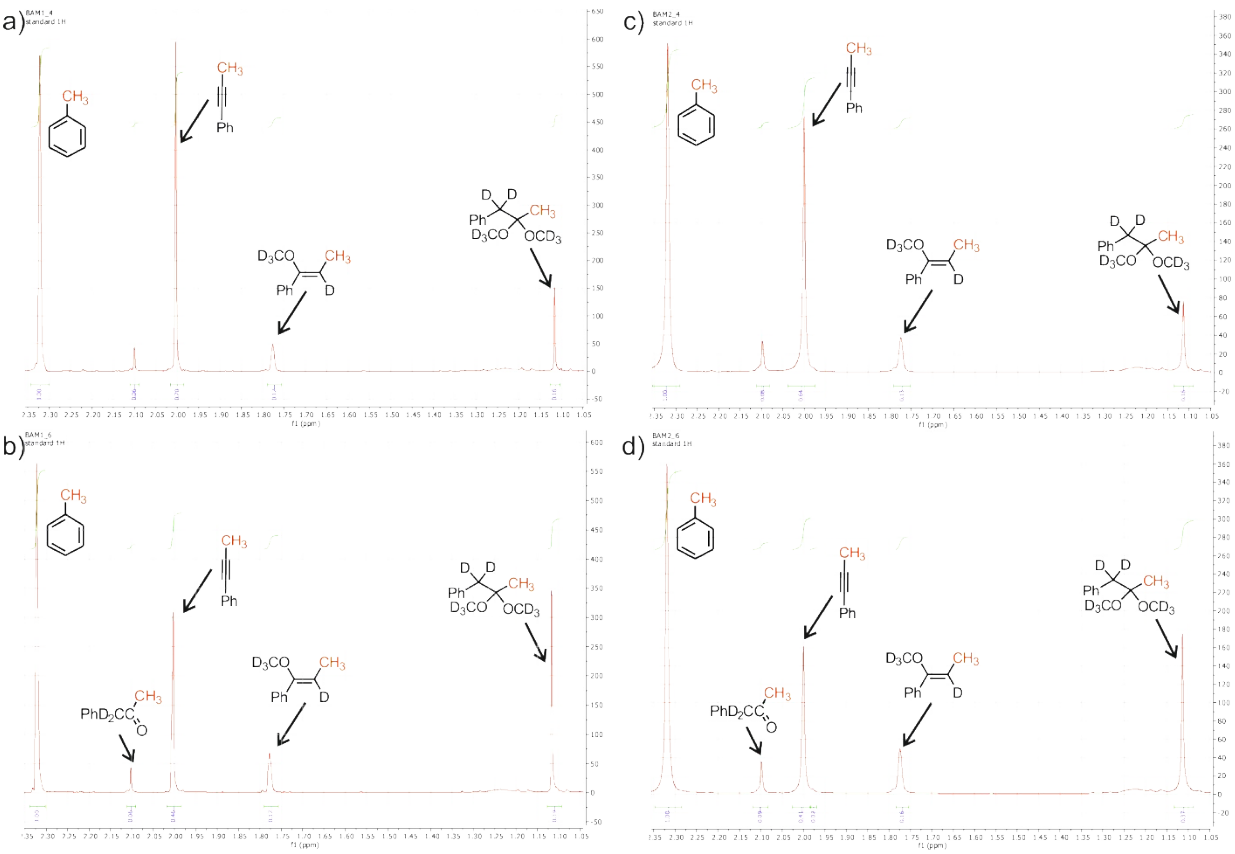

Figure S10. ^{1}H NMR spectra of the reaction mixture of 1.25 mol% [AuCl(IPr)]/1.5 mol% [AgOTf] a) 13 and b) 22 minutes after the addition of the solution of catalyst to the solution of $PhCCCH_3$ and toluene in CD_3OD/CD_2Cl_2 (1:2.4). ^{1}H NMR spectra of the reaction mixture of 1.25 mol% [AuCl(IPr)]/1.5 mol% [AgOTf]/ 1.25 mol% Bn-bambusuril **2** in CD_3OD/CD_2Cl_2 c) 14 and d) 22 minutes after the addition of the solution of catalyst to the solution of $PhCCCH_3$ and toluene in CD_3OD/CD_2Cl_2 (1:2.4). The signals correspond to the methyl groups shown in red in the corresponding formulas.

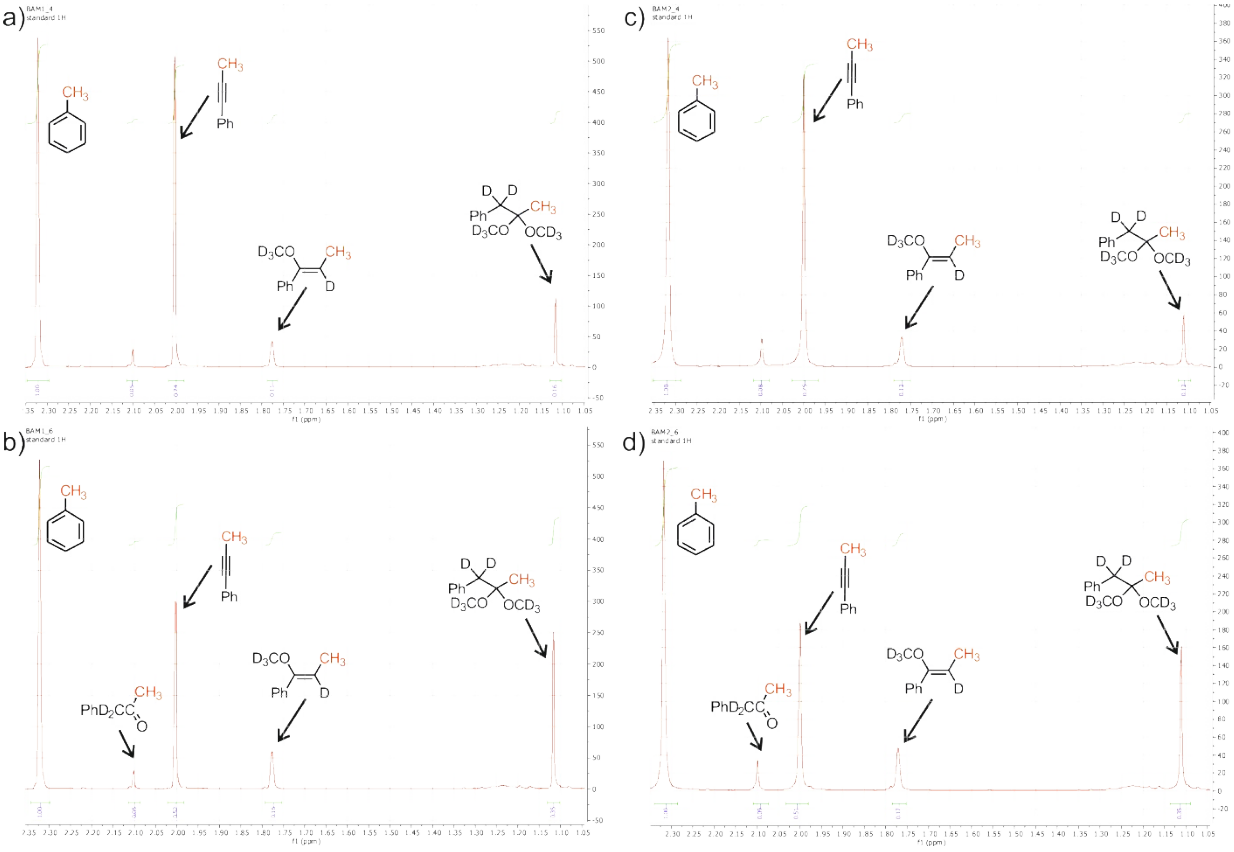

Figure S11. ^{1}H NMR spectra of the reaction mixture of 1.25 mol% [AuCl(IPr)]/1.5 mol% [$AgPF_6$] a) 13 and b) 22 minutes after the addition of the solution of catalyst to the solution of $PhCCCH_3$ and toluene in CD_3OD/CD_2Cl_2 (1:2.4). ^{1}H NMR spectra of the reaction mixture of 1.25 mol% [AuCl(IPr)]/1.5 mol% [$AgPF_6$]/ 1.25 mol% Bn-bambusuril **2** in CD_3OD/CD_2Cl_2 c) 13 and d) 22 minutes after the addition of the solution of catalyst to the solution of $PhCCCH_3$ and toluene in CD_3OD/CD_2Cl_2 (1:2.4). The signals correspond to the methyl groups shown in red in the corresponding formulas.

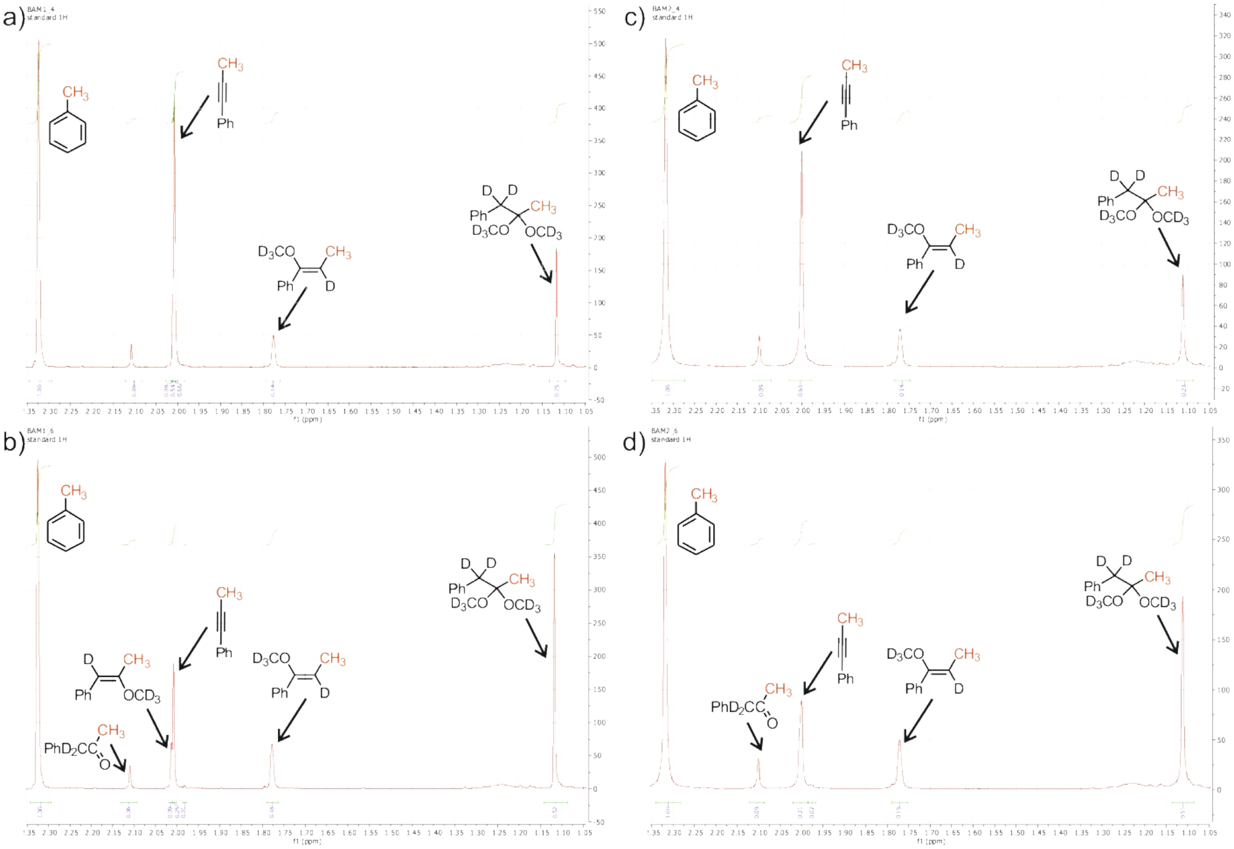

Figure S12. ^{1}H NMR spectra of the reaction mixture of 1.25 mol% [AuCl(IPr)]/1.5 mol% [$AgClO_4$] a) 14 and b) 24 minutes after the addition of the solution of catalyst to the solution of $PhCCCH_3$ and toluene in CD_3OD/CD_2Cl_2 (1:2.4). ^{1}H NMR spectra of the reaction mixture of 1.25 mol% [AuCl(IPr)]/1.5 mol% [$AgClO_4$]/ 1.25 mol% Bn-bambusuril **2** in CD_3OD/CD_2Cl_2 c) 14 and d) 24 minutes after the addition of the solution of catalyst to the solution of $PhCCCH_3$ and toluene in CD_3OD/CD_2Cl_2 (1:2.4). The signals correspond to the methyl groups shown in red in the corresponding formulas.

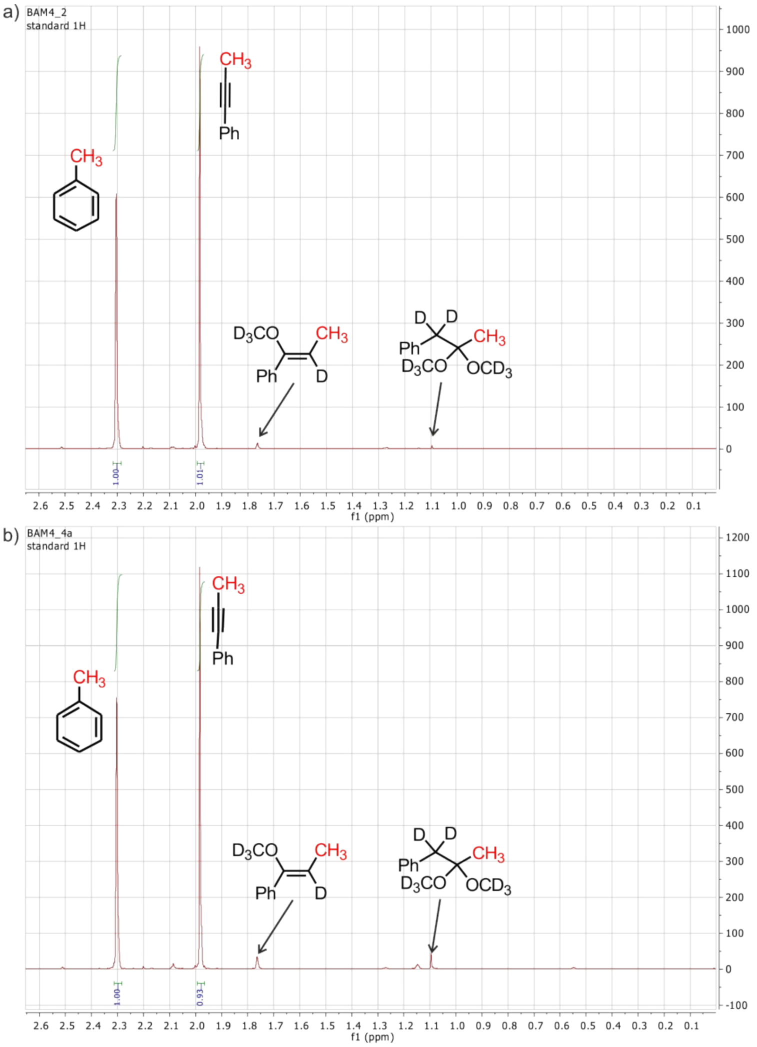

Figure S13. ^{1}H NMR spectrum of the reaction mixture of 1.25 mol% [AuCl(PPh$_3$)]/1.5 mol% [AgSbF$_6$] in CD$_3$OD a) 33 and b) 123 minutes after the addition of PhCCCH$_3$ (details can be found in Material and Methods section). The signals correspond to the methyl groups shown in red in the corresponding formulas.

Preparation of reaction mixtures for mass spectrometry experiments

I) Different concentration of 1-phenylpropyne

The stock solution **A** was prepared by mixing of 4.0 mg of $AgSbF_6$ or 2.9 mg $AgPF_6$ or 3.0 mg AgOTf dissolved in 1.4 ml of CH_3OH and 4.8 mg of $AuCl(PPh_3)$ dissolved in 0.2 ml CH_2Cl_2. The reaction mixture was mixed for 30 minutes and filtered through a PTFE filter (pore size 0.2 µm) to remove precipitated AgCl.

Table S5. Composition of the stock solution **B**

$c_{PhCCCH3}$ [mol.dm^{-3}]	$PhCCCH_3$ [µl]	CH_3OH [ml]
0.015	30	2
0.030	60	2
0.060	120	2
0.090	180	2
0.120	240	2
0.135	270	2
0.150	300	2

Final reaction mixtures were prepared by mixing 80 µl of the stock solution **A**, 80 µl of **B** and 160 µl of CH_3OH (for the composition of the stock solutions see Table S6) and left to react for a time delay 5 minutes. After a time delay elapsed, 320 µl of solution CD_3OH was added. Reaction mixture was immediately monitored by ESI-MS.

II) Different concentration of *p*-toluenesulfonic acid (TsOH)

The stock solution **A** was prepared by mixing of 4.0 mg of $AgSbF_6$ dissolved in 1.4 ml of CH_3OH and 4.8 mg of AuCl(PPh_3) dissolved in 0.2 ml CH_2Cl_2. The reaction mixture was mixed for 30 minutes and filtered through a PTFE filter (pore size 0.2 μm) to remove precipitated AgCl.

Stock solution **B** was prepared by mixing of **120** μl of phenylpropyne and **2** ml of CH_3OH.

Stock solution of *p*-toluenesulfonic acid (TsOH) was prepared by dissolving of **3.8** mg of TsOH in **1.6** ml CH_3OH.

Table S6. Composition of the final solution

Au(PPh_3)Cl:TsOH	1:0.5	1:1	1:2	1:4
Stock **A**	80 μl	80 μl	80 μl	80 μl
Stock **B**	80 μl	80 μl	80 μl	80 μl
Stock TsOH	20 μl	40 μl	80 μl	160 μl
CH_3OH	140 μl	120 μl	80 μl	x
CD_3OH	320 μl	320 μl	320 μl	320 μl

Final reaction mixtures were prepared by mixing the stock solutions **A, B** and TsOH (for the composition of the stock solutions see Table S7) and left to react for a time delay 5 minutes. After a time delay elapsed, 320 μl of solution CD_3OH was added. Reaction mixture was immediately monitored by ESI-MS.

III) Different concentration of iPr-BU*H_2SO_4

The stock solution **A** was prepared by mixing of 4.0 mg of $AgSbF_6$ or 2.9 mg $AgPF_6$ or 3.0 mg AgOTf dissolved in 1.4 ml of CH_3OH and 4.8 mg of $AuCl(PPh_3)$ dissolved in 0.2 ml CH_2Cl_2. The reaction mixture was mixed for 30 minutes and filtered through a PTFE filter (pore size 0.2 μm) to remove precipitated AgCl.

Stock solution **B** was prepared by mixing of **60** μl of phenylpropyne and **1.5** ml of CH_3OH.

Stock solution of iPr-BU*H_2SO_4 was prepared by dissolving of **5.2** mg of **1** in **0.3** ml of CH_3OH.

Table S7. Composition of the final solution

$Au(PPh_3)Cl$: iPr-BU*H_2SO_4	1:0.5	1:1	1:1.5	1:2
Stock **A**	75 μl	75 μl	75 μl	75 μl
Stock **B**	113 μl	113 μl	113 μl	113 μl
Stock **1**	19 μl	37 μl	56 μl	75 μl
CH_3OH	93 μl	75 μl	56 μl	37 μl
CD_3OH	300 μl	300 μl	300 μl	300 μl

Final reaction mixtures were prepared by mixing the stock solutions **A, B** and **1** (for the composition of the stock solutions see Table S8) and left to react for a time delay 5 minutes. After a time delay elapsed, 300 μl of solution CD_3OH was added. Reaction mixture was immediately monitored by ESI-MS.

Delayed reactant labelling method

The method is based on the monitoring of the reaction mixture that contains one of the reactants as a mixture of isotopically labeled and unlabeled molecules in time. In mass spectrum we can see couple of peaks that contain reactant. One of these peaks contains labeled reactant and the other peaks contain unlabeled reactant. The key trick is the time delay (t_D) for the addition of the labeled reactant to the reaction mixture. This allows us to follow the kinetics of all ions that contain this particular reactant. We assume that labeling does not affect effectivity of ion ionization. We evaluate the signals only relative to each other, thus overall intensity is not important.

$$\text{reactant + catalyst} \underset{k_{-I}}{\overset{k_I}{\rightleftarrows}} \text{intermediate} \xrightarrow{k_{II}} \text{product}$$

Scheme S3. Model reaction for a kinetic model derivation

Kinetic model for mathematic description of the experimental data was derived from a model shown in Scheme S3. Here we assume first-order reaction between a reactant (React) and a catalyst (Cat) yielding an intermediate (Int), which then converts on a product (Prod). If formation and degradation of the intermediate (Int) can be described using steady-state approximation, then we can assemble following equation:

$$k_I[\text{React}][\text{Cat}] = \left(k_{-I} + k_{II}\right)[\text{Int}]_{eq} \qquad \text{(S8),}$$

where $[\text{Int}]_{eq}$ is the equilibrium concentration of the intermediate.

The reaction mixture is first prepared with unlabeled reactants and allowed to react for the t_D. During this time a certain concentration of intermediate $[\text{Int}]_{eq}$ is achieved. After time delay a labeled reactant (React^{label}) is added and system is deflected from the steady-state conditions. After addition of a labeled reactant (React^{label}), reaction mixture is monitored by ESI-MS.

The evolution of the intensity of signals that corresponds to the reaction intermediate (Int) and to the labeled intermediate (Int^{label}) in time reflects reestablishment of the steady-state conditions and can be described by following equation:

$$\frac{d[\mathrm{Int}]}{dt} = (k_{-\mathrm{I}} + k_{\mathrm{II}})[\mathrm{Int}]_{\mathrm{eq}} - (k_{-\mathrm{I}} + k_{\mathrm{II}})[\mathrm{Int}] = k'([\mathrm{Int}]_{\mathrm{eq}} - [\mathrm{Int}]), \qquad \text{(S9)},$$

where $k_{\mathrm{MS}} = k_{-\mathrm{I}} + k_{\mathrm{II}}$.

At the mixing time t_0 no labeled intermediate is present in the reaction mixture ($[\mathrm{Int}^{label}] = 0$). If we normalize a sum of concentration of the labeled and unlabeled intermediate to one ($[\mathrm{Int}^{label}] + [\mathrm{Int}] = 1$), their time dependence can be described by following equations:

$$[\mathrm{Int}]_t = e^{-k_{MS}t} + [\mathrm{Int}]_{\mathrm{eq}}(1 - e^{-k_{MS}t}) \qquad \text{(S10a)}$$

$$[\mathrm{Int}^{\mathrm{label}}]_t = [\mathrm{Int}^{\mathrm{label}}]_{\mathrm{eq}}\left(1 - e^{-k_{MS}t}\right). \qquad \text{(S10b)}$$

In the real experiment a precise ratio of the labeled and unlabeled intermediates is obtain by fitting experimental data. For the half-life of the intermediates the following equation can be used:

$$t_{1/2} = \frac{\ln 2}{k_{MS}}. \qquad \text{(S11)}$$

The intermediates formation rate (k_I) does not influence a shape of the curves if we assume that the rate constant for the formation of labeled and unlabeled intermediates is the same. Further we assume that labeled and unlabeled intermediates react with the same rate constant.

Results of mass spectrometry kinetic experiments

Table S8. Half-lives of intermediates $[Au_2(PPh_3)_2(PhCCCH_3,CH_3O)]^+$ (**2**) determined by Delayed Reactant Labelling experiments (the labelled reactant was CD_3OH, and the time delay was 5 min).[a]

X^-	*c* ($PhCCCH_3$) [$mol.dm^{-3}$]	$Au(PPh_3)Cl$ [mol%]	TsOH[b] [mol%]	iPr-BU* H_2SO_4 [mol%]	**2** k_{MS} [min^{-1}]	**2** $t_{1/2}$ [min]
SbF_6	0.015	5.00	-	-	0.05 ± 0.01	13.1 ± 2.2
SbF_6	0.030	2.50	-	-	0.07 ± 0.01	10.0 ± 1.7
SbF_6	0.060	1.25	-	-	0.12 ± 0.03	5.8 ± 1.1
SbF_6	0.090	0.94	-	-	0.14 ± 0.02	5.2 ± 0.7
SbF_6	0.120	0.63	-	-	0.14 ± 0.02	5.0 ± 1.1
SbF_6	0.135	0.56	-	-	0.21 ± 0.03	3.4 ± 0.5
SbF_6	0.150	0.50	-	-	0.23 ± 0.01	3.0 ± 0.1
SbF_6	0.060	0.63	-	-	0.16 ± 0.03	4.4 ± 0.8
SbF_6	0.060	0.83	-	-	0.11 ± 0.01	6.2 ± 0.6
SbF_6	0.060	1.66	-	-	0.11 ± 0.01	6.9 ± 0.5
SbF_6	0.060	2.00	-	-	0.14 ± 0.03	5.0 ± 0.9
SbF_6	0.060	2.50	-	-	0.13 ± 0.02	5.4 ± 0.9
SbF_6	0.060	3.13	-	-	0.13 ± 0.01	5.3 ± 0.2

SbF_6	0.060	1.25	0.63	-	0.20 ± 0.06	3.8 ± 1.0
SbF_6	0.060	1.25	1.25	-	0.19 ± 0.05	3.9 ± 1.2
SbF_6	0.060	1.25	2.50	-	0.30 ± 0.02	2.3 ± 0.2
SbF_6	0.060	1.25	5.00	-	0.48 ± 0.09	1.5 ± 0.3
OTf	0.060	1.25	-	-	0.13 ± 0.02	5.4 ± 0.7
PF_6	0.060	1.25	-	-	0.14 ± 0.04	5.3 ± 1.5
SbF_6	0.060	1.25	-	0.63	0.33 ± 0.13	2.5 ± 1.0
SbF_6	0.060	1.25	-	1.25	0.38 ± 0.16	2.1 ± 0.8
OTf	0.060	1.25	-	1.25	0.25 ± 0.03	2.8 ± 0.4
PF_6	0.060	1.25	-	1.25	0.26 ± 0.09	3.0 ± 1.0
SbF_6	0.060	1.25	-	2.50	0.42 ± 0.10	1.8 ± 0.4

[a] CD_3OH was added to the reaction mixture of different concentration of 1-phenylpropyne with different mol% [Au(PPh_3)Cl]/AgX in methanol.

[b] Different mol% of *p*-toluenesulfonic acid (TsOH) was added to the reaction mixture

[c] Different mol% of Pr-BU*H_2SO_4 was added to the reaction mixture.

Synthetic protocols and characterization of bambusurils

1,3-Diisopropyl urea

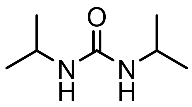

Diphenyl carbonate (9.97 g, 46.6 mmol) was suspended in isopropyl amine (16.0 mL, 186.2 mmol, 4.0 eq.) cooled to 0 °C. The mixture was heated in MW reactor (100 °C, 50 W) for 2.15 h. Reaction mixture was diluted with water (15 ml). The mixture was filtered, washed with cold H_2O (3×50 ml), ice-cold Et_2O (50 ml), and dried to obtain 5.32 g of white product in quantitative yield.

^{1}H NMR (500 MHz, DMSO-d_6) δ 5.47 (d, J = 7.8 Hz, 2H), 3.64 (m, 2H), 1.00 (d, J = 6.5 Hz, 12H).

^{13}C NMR (126 MHz, DMSO-d_6) δ 156.75, 40.63, 23.24.

HRMS (APCI+): m/z calcd for [$C_7H_{16}N_2O+H^+$]: 145.1335, found: 145.1334.

2,4-Diisopropyl glycoluril

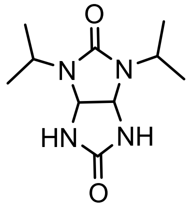

1,3-Diisopropyl urea (10.00 g, 69.34 mmol) was dissolved in mixture of MeOH (100 mL) and conc. HCl (2.0 mL), and heated to 80 °C. 4,5-Dihydroxyimidazolidin-2-one (10.24 g, 86.68 mmol, 1.25 eq.) was added in two portions over 1 h. The warm reaction mixture was filtered after 6 h of heating to obtain 8.96 g of material containing traces of unsubstituted glycoluril. The impurity was removed by reflux in water (100 ml) and filtration to give product in yield 8.00 g (51 %).

^{1}H NMR (500 MHz, DMSO-d_6) δ 7.39 (s, 2H), 5.27 (d, J = 1.0 Hz, 2H), 3.95 – 3.68 (m, 2H), 1.10 (dd, J = 6.9, 5.5 Hz, 12H).

^{13}C NMR (126 MHz, DMSO-d_6) δ 161.46, 156.65, 64.04, 43.35, 21.49, 19.00.

HRMS (APCI+): m/z calcd for [$C_{10}H_{18}N_4O_2+H^+$]: 227.1503, found: 227.1501.

iPr-BU*H_2SO_4

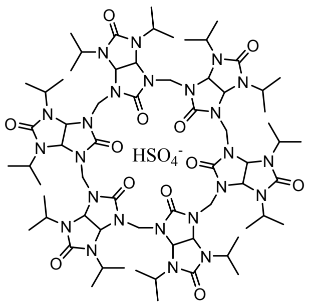

2,4-Diisopropyl glycoluril (7.00 g, 30.94 mmol) and paraformaldehyde (1.16 g, 38.67 mmol, 1.25 eq.) were heated in dioxane (140 mL) to 80 °C. Conc. H_2SO_4 (4.2 mL) was added and the reaction mixture was heated for 2.5 hours. The reaction mixture was cooled down to RT and diluted with H_2O (50 ml). Solid material was collected by filtration, washed twice with H_2O (50 ml) and dried under vacuum. Raw product was dissolved in MeOH and filtered. The filtrate was evaporated to yield 5.68 g (77 %) of $iPr_{12}BU[6]\cdot HSO_4^-$ (H^+) complex.
^{1}H NMR (500 MHz, DMSO-d_6) δ 5.40 (s, 12H), 4.79 (s, 12H), 4.23 (m, 12H), 1.37 (d, J = 6.9 Hz, 36H), 1.20 (d, J = 6.6 Hz, 36H).
^{13}C NMR (126 MHz, DMSO-d_6) δ 159.06, 158.19, 68.55, 47.28, 20.87, 19.14.
HRMS (APCI-): m/z calcd for [$C_{66}H_{109}N_{24}O_{16}S+Cl^-$]: 1463.8273, found: 1463.8297.

Anion-free iPr-BU

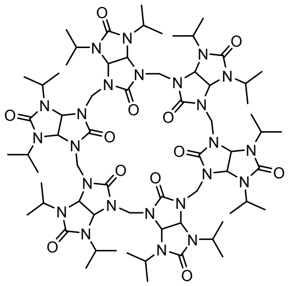

The iPr-BU*H_2SO_4 complex (1.50 g) was refluxed for 1 hour with 25% aq. NH_3 (1.5 ml) in mixture of H_2O(10 ml, mili-Q) and MeOH (10 ml). The insoluble solid was collected by filtration, washed with mili-Q water (2×) and MeOH, and dried to obtain anion-free dodecaisopropylbambus[6]uril in quantitative yield (1.40 g).
^{1}H NMR (500 MHz, Chloroform-d) δ 5.45 (s, 12H), 4.76 (s, 12H), 1.49 (d, J = 7.0 Hz, 36H), 1.33 (d, J = 6.8 Hz, 36H).
^{13}C NMR (126 MHz, Chloroform-d) δ 159.56, 158.88, 70.24, 48.92, 48.70, 20.65, 19.95.
HRMS (APCI+): m/z calcd for [$C_{66}H_{108}N_{24}O_{12}+H^+$]: 1429.8651, found: 1429.8653.

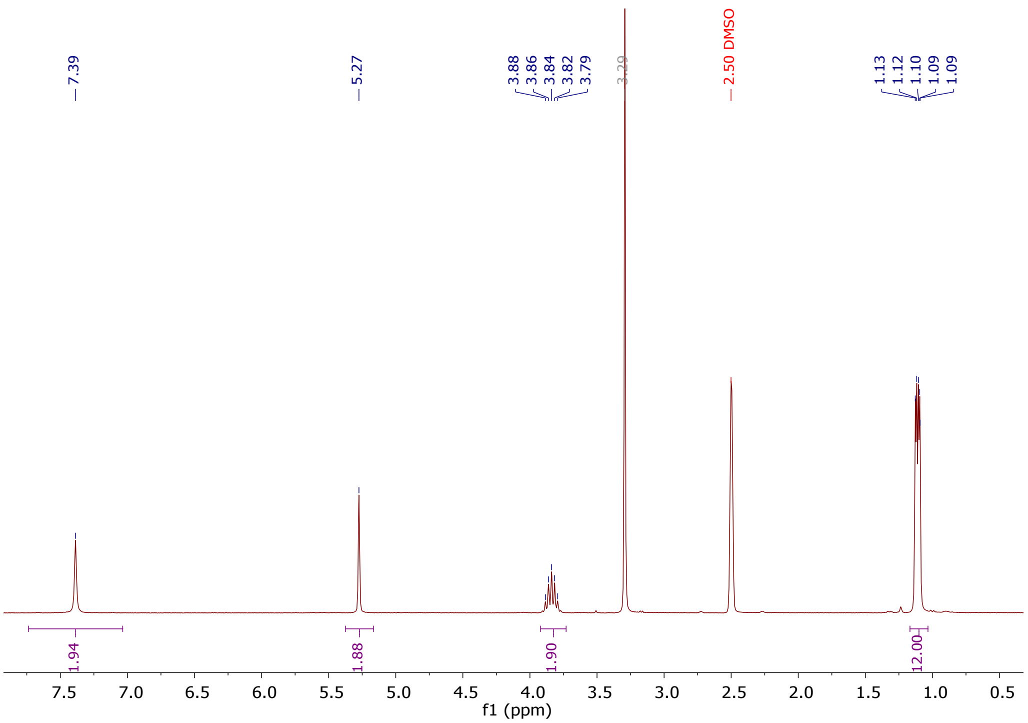

Figure S14: ^{1}H NMR spectrum of 2,4-diisopropylglycoluril.

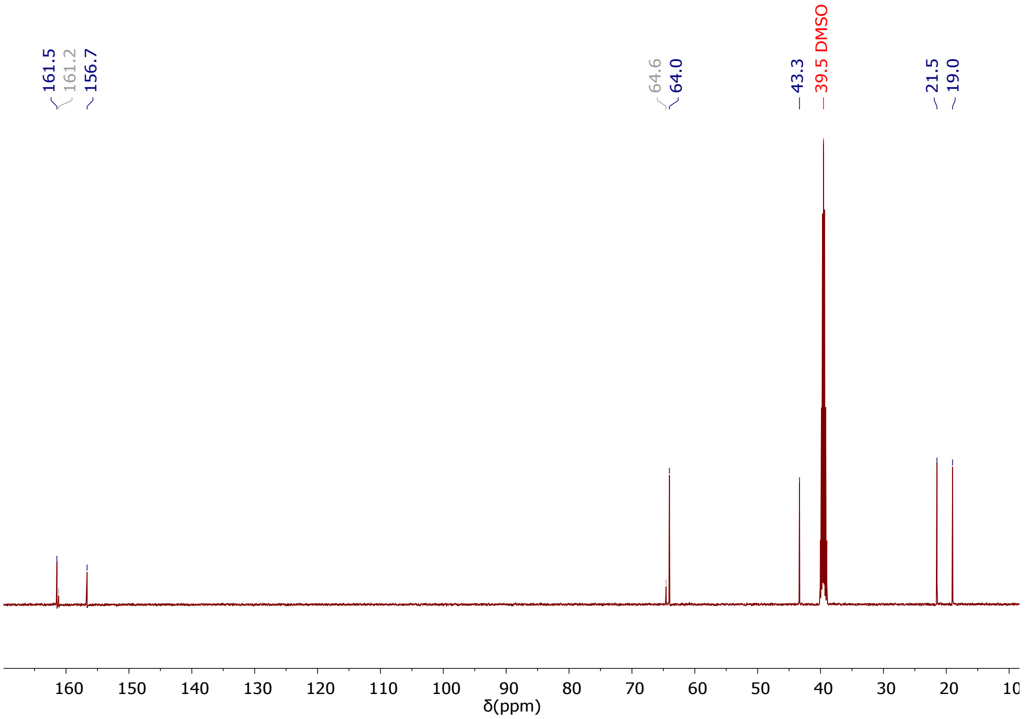

Figure S15: ^{13}C NMR spectrum of 2,4-diisopropylglycoluril.

.

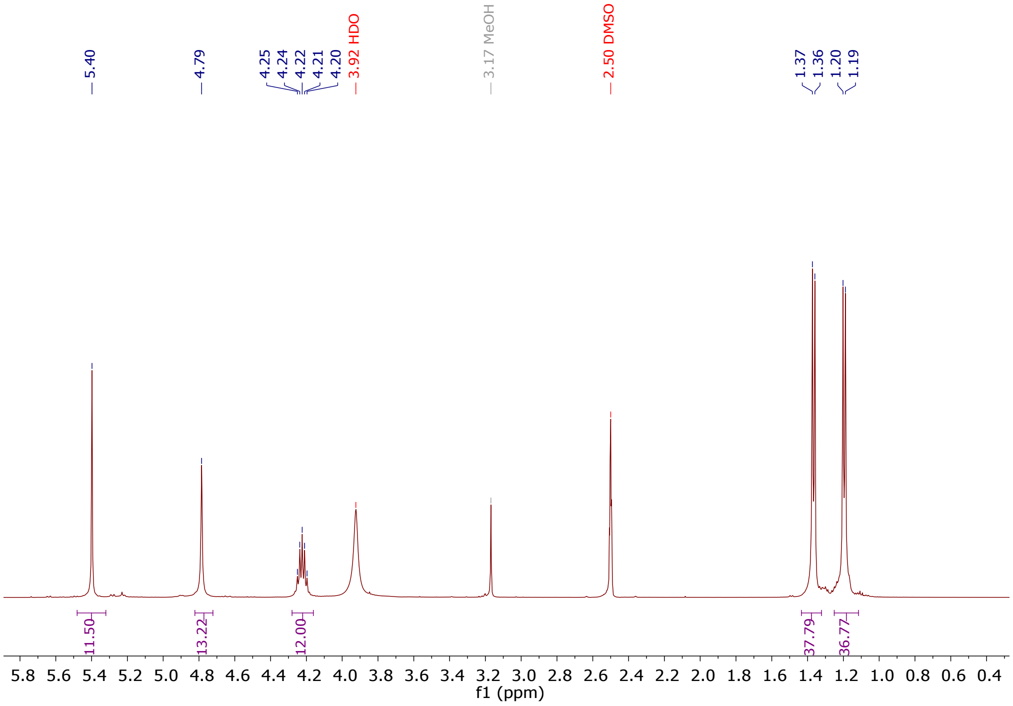

Figure S16: ^{1}H NMR spectrum of iPr-BU*H_2SO_4.

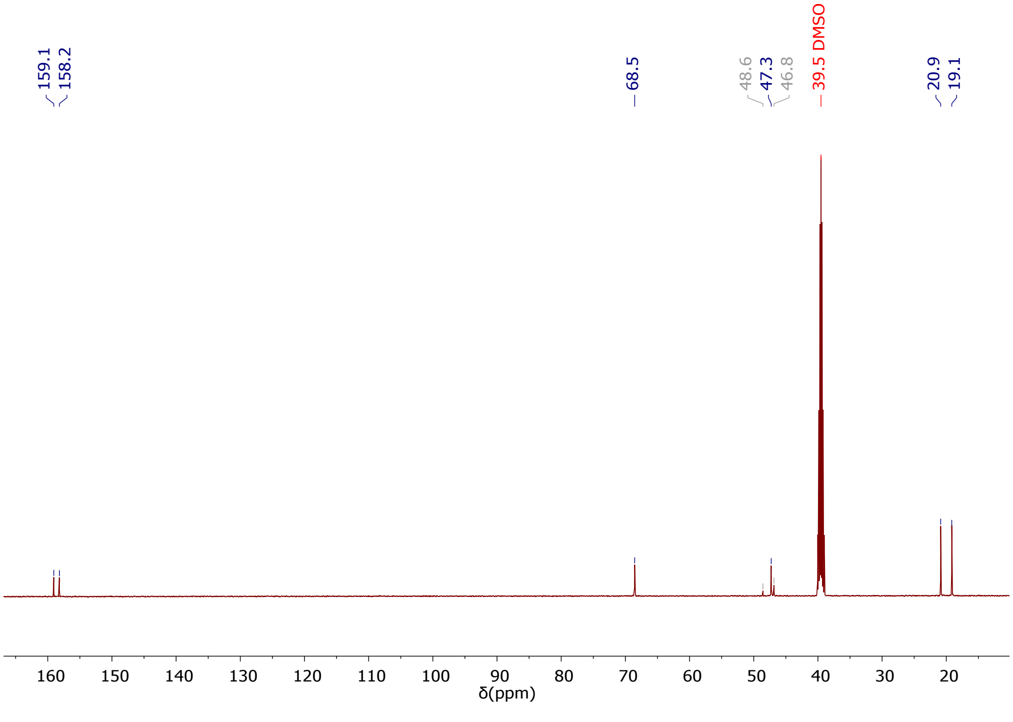

Figure S17: ^{13}C NMR spectrum of iPr-BU*H_2SO_4.

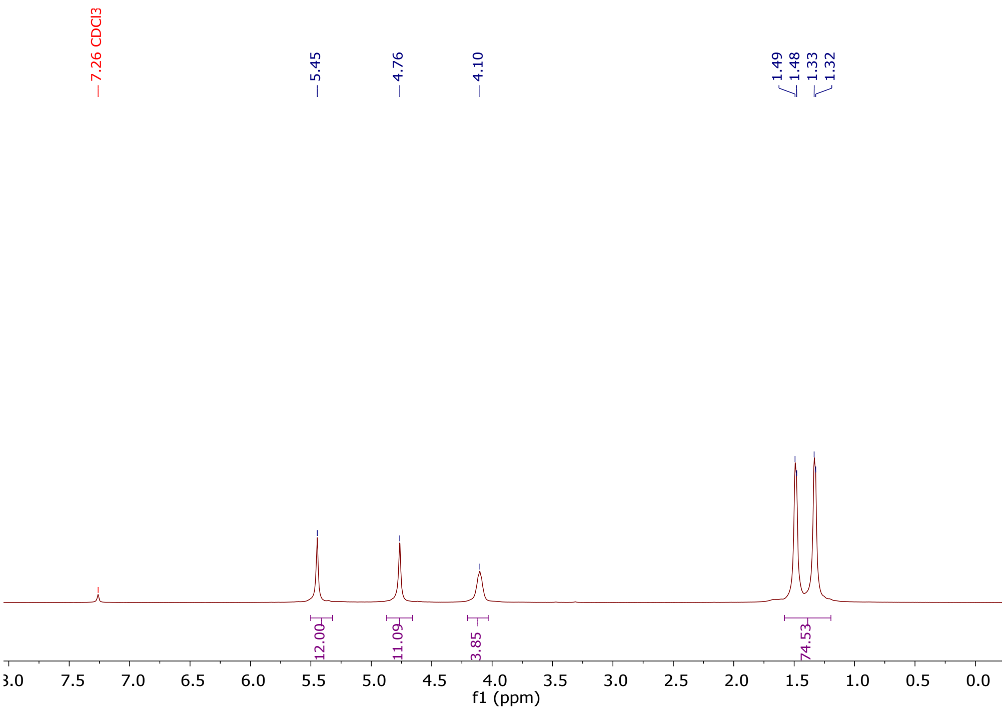

Figure S18: ^{1}H NMR spectrum of anion-free iPr-BU.

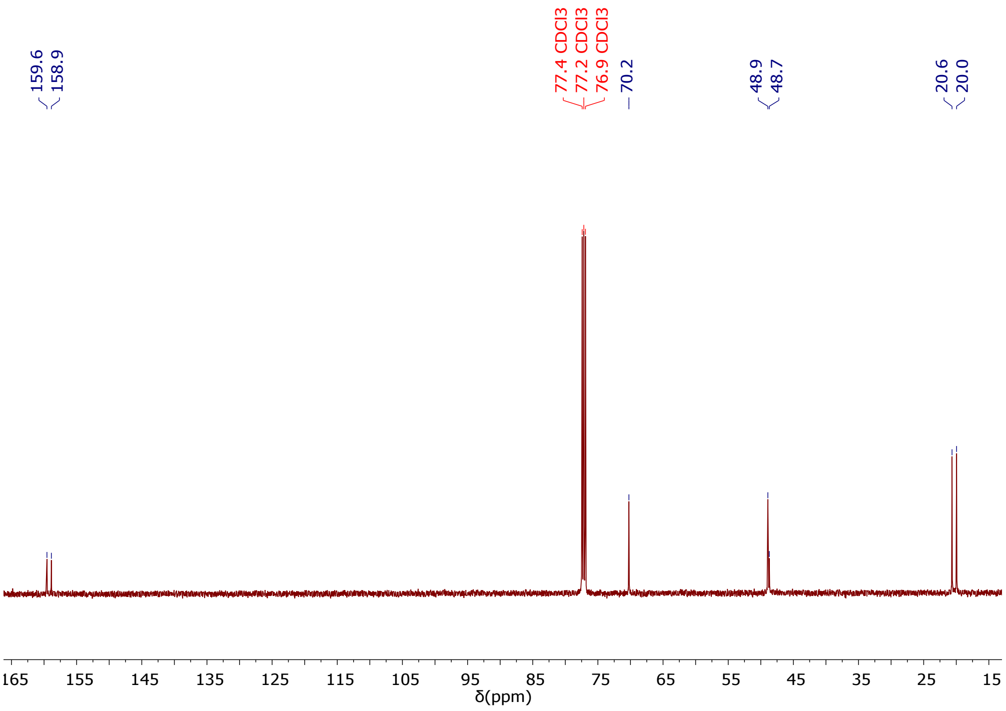

Figure S19: ^{13}C NMR spectrum of anion-free iPr-BU.

NMR competition experiments

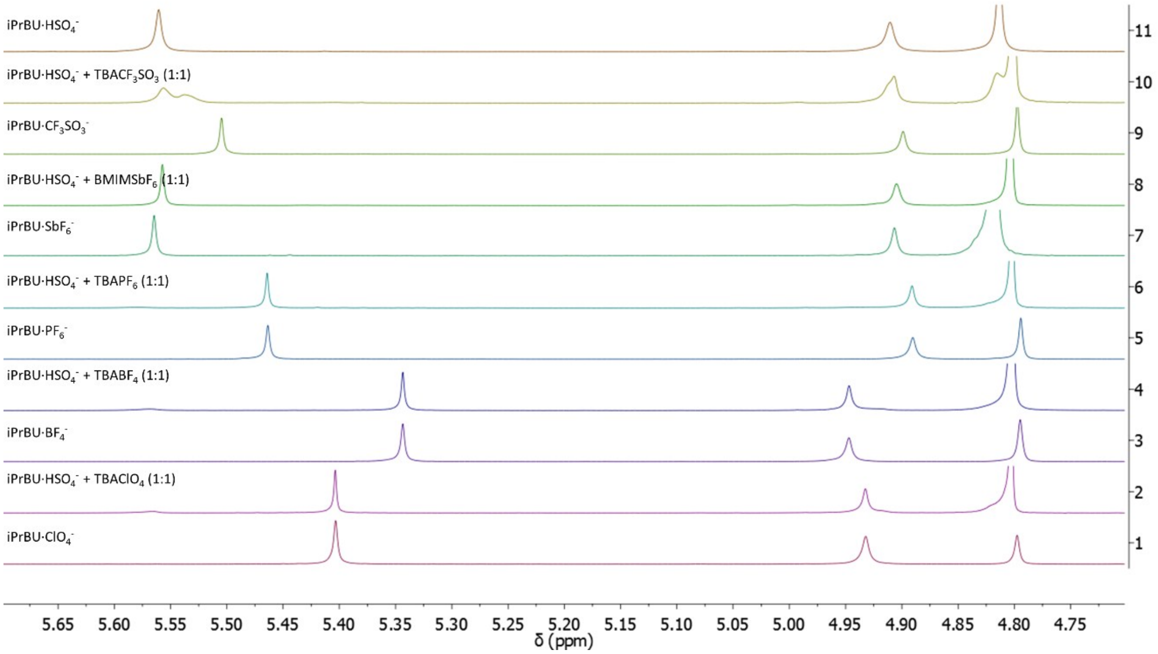

Figure S20: ^{1}H NMR spectra of iPrBU*H_2SO_4 in the presence of 0.5 equivalent (unless stated otherwise) of $CF_3SO_3^-$, SbF_6^-, PF_6^-, BF_4^-, and ClO_4^-, compared to the spectra of iPrBU complexes with corresponding anions. All spectra measured in CD_3OD at 30°C. TBA = tetrabutylammonium, BMIM = 1-butyl-3-methylimidazolium.

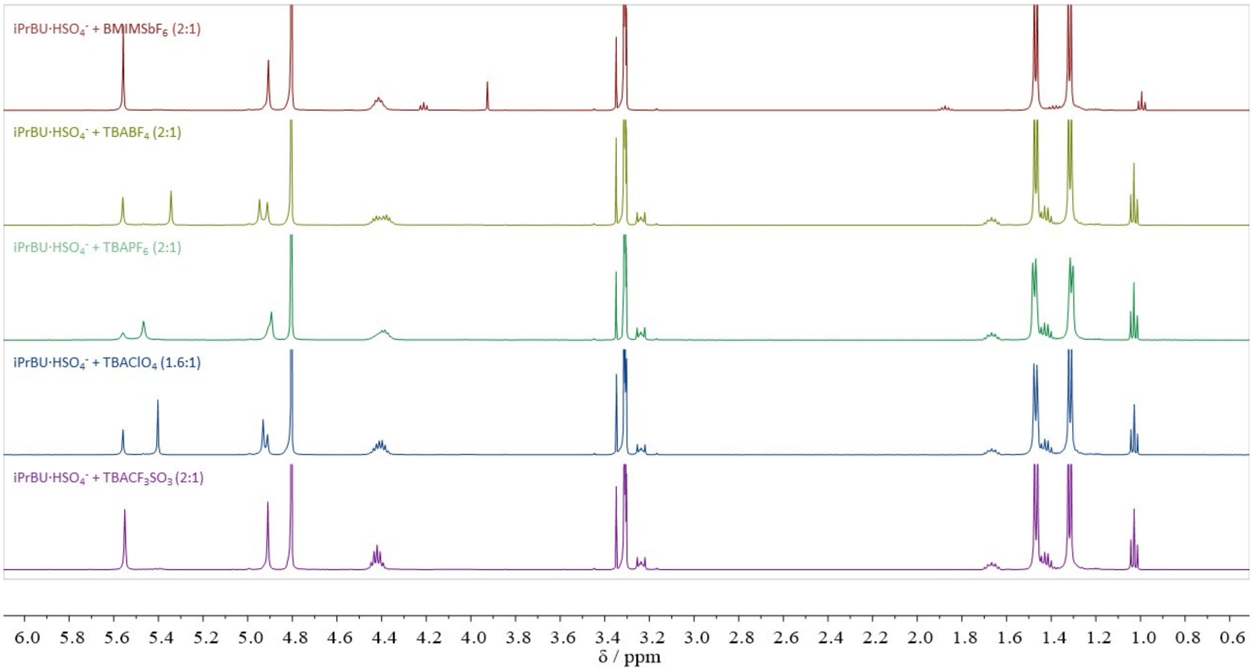

Figure S21: ^{1}H NMR spectra of iPrBU*H_2SO_4 in the presence of 0.5 equivalent (unless stated otherwise) of $CF_3SO_3^-$, SbF_6^-, PF_6^-, BF_4^-, and ClO_4^-. All spectra measured in CD_3OD at 30°C. TBA = tetrabutylammonium, BMIM = 1-butyl-3-methylimidazolium.
